# Size Control and Fluorescence Labeling of Polydopamine Melanin-Mimetic Nanoparticles for Intracellular Imaging

**DOI:** 10.3390/biomimetics2030017

**Published:** 2017-09-06

**Authors:** Devang R. Amin, Caroline Sugnaux, King Hang Aaron Lau, Phillip B. Messersmith

**Affiliations:** 1 Departments of Bioengineering and Materials Science and Engineering, University of California, Berkeley, 210 Hearst Mining Building, Berkeley, CA 94720, USA; damin@berkeley.edu (D.R.A.); csugnaux@berkeley.edu (C.S.); 2 Department of Biomedical Engineering, Northwestern University, 2145 Sheridan Rd., Evanston, IL 60208, USA; 3 WestCHEM/Department of Pure and Applied Chemistry, University of Strathclyde, 295 Cathedral St., Glasgow G1 1XL, UK; aaron.lau@strath.ac.uk

**Keywords:** catechol, melanin, nanoparticle, dopamine

## Abstract

As synthetic analogs of the natural pigment melanin, polydopamine nanoparticles (NPs) are under active investigation as non-toxic anticancer photothermal agents and as free radical scavenging therapeutics. By analogy to the widely adopted polydopamine coatings, polydopamine NPs offer the potential for facile aqueous synthesis and incorporation of (bio)functional groups under mild temperature and pH conditions. However, clear procedures for the convenient and reproducible control of critical NP properties such as particle diameter, surface charge, and loading with functional molecules have yet to be established. In this work, we have synthesized polydopamine-based melanin-mimetic nanoparticles (MMNPs) with finely controlled diameters spanning ≈25 to 120 nm and report on the pH-dependence of zeta potential, methodologies for PEGylation, and the incorporation of fluorescent organic molecules. A comprehensive suite of complementary techniques, including dynamic light scattering (DLS), cryogenic transmission electron microscopy (cryo-TEM), X-ray photoelectron spectroscopy (XPS), zeta-potential, ultraviolet*–*visible (UV–Vis) absorption and fluorescence spectroscopy, and confocal microscopy, was used to characterize the MMNPs and their properties. Our PEGylated MMNPs are highly stable in both phosphate-buffered saline (PBS) and in cell culture media and exhibit no cytotoxicity up to at least 100 µg mL^−1^ concentrations. We also show that a post-functionalization methodology for fluorophore loading is especially suitable for producing MMNPs with stable fluorescence and significantly narrower emission profiles than previous reports, suggesting they will be useful for multimodal cell imaging. Our results pave the way towards biomedical imaging and possibly drug delivery applications, as well as fundamental studies of MMNP size and surface chemistry dependent cellular interactions.

## 1. Introduction

Nanotechnology has garnered tremendous attention from the biomedical community over the past decade due to its potential to revolutionize cancer treatment by delivering targeted packages of chemotherapeutic drugs, thereby minimizing their adverse side-effects and boosting bioavailability [1,2,3,4,5,6,7,8]. Despite intense research, however, few nanotechnology-based solutions are clinically-approved as cancer therapeutics [8,9]. An improved understanding of nanoparticle–cell and nanoparticle–body interactions is essential for the optimization of nanoparticle (NP) design to improve therapeutic outcomes. Fluorescent NPs enable in-depth study of these phenomena, as illustrated in the recent use of quantum dots (QDs) by Chan et al. to study the fundamental mechanisms of hard NP clearance by the liver [10]. Improving the design of organic NP-based therapeutics requires study of soft organic NPs rather than hard inorganic NPs like QDs, spurring interest in fluorescent organic NPs (FONs) [11,12]. A variety of approaches including emulsion polymerization, block copolymer self-assembly, and nanoprecipitation in the presence of a fluorophore have been employed in the synthesis of FONs [11,12]. However, many FON synthesis techniques require the use of toxic organic solvents or surfactants, which must be removed following synthesis.

The recent development of polydopamine melanin-like NPs has created an opportunity to generate organic NPs in a non-toxic, straightforward strategy. Inspired by the presence of sepia melanin NPs in cuttlefish ink, Ju et al. first reported a synthesis of non-toxic melanin-like NPs composed of polydopamine [13]. Nanoparticles were prepared by dissolving dopamine·HCl in water at basic pH to generate polydopamine NPs, which had melanin-like free radical scavenging activity. Although the exact mechanism and species involved in the formation of polydopamine are still actively under investigation, there is mounting experimental and computational evidence that polydopamine forms via oxidation of dopamine, producing a complex series of subsequent reactions that are not fully understood [14,15]. This material has properties similar to the biological pigment eumelanin, which has functions including protection against harmful ultraviolet (UV) light, free radical scavenging, heavy metal sequestration, and structural roles, as in the *Glycera dibranchiata* bloodworm jaw [16,17,18,19].

One unique property of polydopamine is its chemical versatility. Studies on polydopamine surface coatings have shown that it can subsequently be modified via covalent bonding with amines and thiols, hydrogen bonding, π–π stacking, metal coordination, and electrostatic interactions [20,21]. This characteristic of polydopamine may be leveraged to form multifunctional polydopamine-based melanin-mimetic NPs (MMNPs) for biomedical applications without the use of coupling reagents or biomolecular modification, unlike many existing fluorescent NP systems [11,12,22].

Since the initial synthesis of MMNPs by Ju et al. [13], alternate methods of creating MMNPs have also been developed [23,24]. Polydopamine NPs have shown promising results when studied as melanin-like UV-protective materials in cells [25], anticancer photothermal agents in vivo [23], and as magnetic resonance imaging (MRI) contrast agents [26]. Despite these reports, neither the reproducibility of NP size control over a broad range of sub-200 nm diameters nor the NP surface charge have yet been studied in depth.

Related work has focused on synthesis of fluorescent microcapsules or plate-like nanostructures of polydopamine via oxidation by H_2_O_2_ [26,27,28] or by combination of polyethyleneimine and polydopamine [29,30]. These novel nanomaterials have promise as potential fluorescent organic NPs or microparticles, but they possess several shortcomings. First, the sizes and morphologies of these fluorescent materials limit conclusions that could be drawn with regard to the uptake and trafficking of spherical NPs with diameters below 100–200 nm, which is the most relevant size ranges for injectable nanotherapeutics. Second, each of these published methods of fluorescent polydopamine preparation results in broad fluorescence excitation and emission spectral peaks that may interfere with dyes to be used as co-stains for in vitro studies. Full-width half maximum (FWHM) of the peaks in these studies are on the order of 100 nm, limiting simultaneous use of other fluorophores. In contrast, quantum dots have spectral linewidths of just 12 nm [31]. Third, fluorescence excitation and emission peaks cannot be tuned using this approach. Modification of NPs with different fluorophores would permit this fine-tuning. We sought to address these three issues through our research.

In this work, we demonstrate a novel, straightforward method by which MMNPs with reproducibly tunable diameters under 100–200 nm can be synthesized and modified by two fluorescent rhodamine dyes, rhodamine 123 (RA123) and rhodamine B (RAB). We have developed procedures by which MMNPs may be rhodamine-labeled either in situ during MMNP formation or by post-functionalization of PEGylated MMNPs. Neither of these methods require any toxic or expensive chemical coupling reagents, organic solvents, surfactants, or oxidizing agents. We demonstrate that these materials have narrower fluorescence excitation and emission peaks (FWHM ≈40 nm) relative to other methods of fluorescent polydopamine NP preparation. As a proof of concept, we show that PEGylated fluorescent MMNPs are taken up by cells and accumulate in the perinuclear region, where they can be visualized by confocal microscopy.

## 2. Materials and Methods

### 2.1. Materials

Dopamine·HCl (DA, >98% purity), rhodamine 123 (RA123), and rhodamine B base (RAB) were purchased from Sigma-Aldrich (St. Louis, MO, USA). Methyl ether poly(ethylene glycol)-thiol (5 kDa, mPEG-SH) was purchased from Laysan Bio, Inc. (Arab, AL, USA). Neutral red dye was purchased from Amresco (Solon, OH, USA). Ultrapure (UP) water was obtained by purification of deionized water with a Barnstead Ultrapure Water Purification System (Thermo Fisher Scientific, Waltham, MA, USA) to a resistivity of at least 18.0 MΩ cm. Amicon® Ultra centrifugal filters with 10 kDa and 100 kDa molecular weight cut-off (MWCO) were obtained from EMD Millipore (Billerica, MA, USA). Dialysis cassettes (10 kDa MWCO) and cell culture reagents were obtained from Thermo Fisher Scientific. NIH/3T3 fibroblasts were purchased from the American Type Culture Collection (ATCC, Manassas, VA, USA).

### 2.2. Nanoparticle Synthesis and Modification

#### 2.2.1. MMNP Synthesis

Melanin-mimetic nanoparticle synthesis was adapted from Ju et al. [13]. For a typical synthesis (1:1 NaOH:DA, 1 mg mL^−1^ DA), 22.68 mL of UP water and 1.32 mL of 0.1 M NaOH were added to a 50 mL round bottom flask and heated to 50 °C under vigorous stirring. Then, 1 mL of a 25 mg mL^−1^ DA solution was added. The flask was tightly capped, and the solution was vigorously stirred for 5 h at 50 °C. After 5 h, the reaction mixture was purified by centrifugal filtration (10 kDa MWCO), washing with UP water. Then, aggregates were removed by centrifugation at between 2000 and 6000 g followed by 0.45 μm filtration. The hydrodynamic diameters (D_h_) of MMNPs were adjusted by controlling DA concentration (1 to 4 mg mL^−1^) and NaOH:DA molar ratio (0.5:1 to 1:1).

#### 2.2.2. In Situ Modification of MMNPs with Rhodamine B or Rhodamine 123

For in situ fluorophore modification, MMNPs synthesis was performed as noted above in a growth solution of 1 mg mL^−1^ DA and 1:1 NaOH:DA supplemented with 50 μg mL^−1^ RAB or RA123.

#### 2.2.3. PEGylation of MMNPs

Melanin-mimetic nanoparticles were treated with 10 mM 5 kDa mPEG-SH overnight in 10 mM NaOH. Unbound mPEG-SH was removed by centrifugal filtration (100 kDa MWCO) at 2000× *g* with washing.

#### 2.2.4. Post-Functionalization of MMNP@PEG with Rhodamine B or Rhodamine 123

Purified MMNP@PEG were post-functionalized for 24 h with RAB or RA123 in either UP water or aqueous pH 8.5 bicine buffer containing 40 µg mL^−1^ MMNP@PEG and 50 µg mL^−1^ RA123 or RAB. The post-functionalized NPs (MMNP@PEG@RAB and MMNP@PEG@RA123) were initially purified by at least six rounds of centrifugal filtration (100 kDa MWCO) with washing to remove unbound fluorophore. Nanoparticles were dialyzed (10 kDa MWCO) for four days in UP water before use in cell culture, replacing the dialysis bath at least five times.

#### 2.2.5. Fluorophore Release Testing

In order to evaluate the release of fluorophore, 50–100 µg fluorophore-labeled MMNPs were dialyzed (10 kDa MWCO) in 200 mL UP water or 1× phosphate-buffered saline (PBS). MMNP@RA123@PEG and MMNP@RAB@PEG were dialyzed for seven days in PBS, and MMNP@PEG@RA123 and MMNP@PEG@RAB were dialyzed sequentially in UP water for three days and in 1× PBS for three days. The dialysis baths were replaced every 4–6 h for the first 12 h to preserve sink conditions and at least every 24 h for the next several days. Fluorophore release was quantified by measuring fluorescence of aliquots of the dialysis baths (Tecan Infinite M200, Männendorf, Switzerland) and quantifying fluorophore content using standard curves prepared from RA123 and RAB stock solutions.

### 2.3. Nanoparticle Characterization

#### 2.3.1. Extinction Coefficient Calculation

An ultraviolet–visible (UV–Vis) plate reader (Synergy H1, BioTek, Winooski, VT, USA) was used to determine absorbances of solutions with known concentrations of MMNPs at wavelengths from 300 to 1000 nm. At least three batches of MMNPs from each set of synthesis conditions was used to calculate the extinction coefficients. Initial concentration of MMNPs was calculated by lyophilizing 1 mL suspensions of MMNPs. Exponential decay curves were fit to extinction coefficient data using OriginPro 2017 software (Student version, OriginLab, Northampton, MA, USA).

#### 2.3.2. Dynamic Light Scattering and Zeta Potential Analysis

Dynamic light scattering (DLS) and zeta potential analysis of NPs was conducted using a Malvern Zetasizer Nano ZS instrument (Malvern, Worcestershire, UK). The z-average NP diameters of NP batches were calculated using cumulants analysis and was reported as the D_h_. The polydispersity index (PDI) was also measured by DLS. During zeta potential measurements, pH was controlled during measurements by using 10 mM citrate buffer (pH 2.5–6.5), 10 mM N-2-hydroxyethylpiperazine-N-2-ethane sulfonic acid (HEPES) buffer (pH 7.0–7.5), or 10 mM bicine buffer (pH 8.0–9.0). Unless otherwise noted, zeta potential was measured at pH 7.4. At least three independently prepared batches of NPs were used for every reported D_h_ or zeta potential value.

#### 2.3.3. Electron Microscopy Imaging

Scanning electron microscopy (SEM) images were obtained using an FEI Quanta 3D FEG SEM (Hillsboro, OR, USA). Conventional transmission electron microscopy (TEM) imaging was performed on a JEOL 1400 TEM (Tokyo, Japan) or FEI Tecnai 12 TEM (Hillsboro, OR, USA). Samples were prepared either with or without uranyl acetate staining. Cryogenic TEM (cryo-TEM) imaging was performed using a JEOL 1230 TEM (Tokyo, Japan). Nanoparticle size analysis was performed using ImageJ software [32] to measure diameters of at least 35 NPs from representative cryo-TEM images of each size range. All NPs in representative images were included in size analysis.

#### 2.3.4. Spectroscopic Characterization

Fluorescence spectra of fluorescent NPs, fluorophores, and unmodified MMNPs were taken using a FluoroMax-4 spectrophotometer (Horiba Scientific, Irvine, CA, USA) with 5 nm slit widths. Samples containing RA123 and RA123 were excited at λ_ex_ = 500 nm, and those containing RAB and RAB were excited at λ_ex_ = 555 nm. The UV–Vis absorbance spectra were taken using a PerkinElmer Lambda UV/Vis/NIR (Waltham, MA, USA) or a UV2600 spectrophotometer (Shimadzu Scientific Instruments, Kyoto, Japan). Fluorescent spectra were not normalized, but absorbance spectra were normalized by multiplying each curve by a constant factor.

#### 2.3.5. X-ray Photoelectron Spectroscopy Characterization

Gold-coated silicon substrates were first cleaned by sonication in UP water, acetone, and isopropanol for 10 min each. Then, after drying them with a flow of nitrogen, the substrates were exposed to a plasma discharge at 60 W for 10 min (Harrick Plasma Cleaner, Ithaca, NY, USA). A 50 µL drop of each NP suspension was then placed onto the surface of the substrates and left to dry overnight. Substrates were completely dried under vacuum prior to analysis using a PHI 5600 spectrometer (PerkinElmer) equipped with an Al monochromated 2 mm filament and a built-in charge neutralizer. The X-ray source operated at 350 W, 14.8 V, and 40**°** take-off angle. The atomic concentrations of sulfur, nitrogen, oxygen, and carbon of drop-casted MMNP and MMNP@PEG samples by performing survey scans between 0 and 1100 eV electron binding energies. Charge correction was performed setting the C 1s peak at 285.0 eV. Data analysis was conducted using MultiPak software version 9.6.015 (Physical Electronics, Chanhassen, MN, USA).

### 2.4. In Vitro Uptake and Cytocompatibility Evaluation

#### 2.4.1. MMNP@PEG Cytocompatibility Study

The procedure used for MMNP@PEG cytocompatibility quantification by neutral red uptake was adapted from Repetto et al. [33]. NIH/3T3 fibroblasts were seeded onto a 96-well plate (10,000 cells/well) and incubated overnight in Dulbecco's Modified Eagle's medium (DMEM, Life Technologies Corporation, Carlsbad, CA, USA) supplemented with 5% newborn calf serum (NBCS, Fisher Scientific, Chicago, IL, USA) and 1% penicillin/streptomycin (Life Technologies Corporation). The cell media were then removed, and 0.2 µm filtered 42, 83, and 146 nm diameter MMNP@PEG samples were introduced to the wells in DMEM supplemented with 5% NBCS with penicillin/streptomycin. Dead cell control wells were treated with 0.2 mg mL^−1^ sodium lauryl sulfate-containing media, and live cell control wells were treated with media without MMNP@PEG. Each treatment was performed in three wells. After incubating the fibroblasts with MMNP@PEG for 24 h, cell media were removed from all wells, and the cells were rinsed with PBS. A 40 µg mL^−1^ neutral red solution was added to the wells in DMEM, and the cells were incubated for 3 h. The DMEM was then aspirated off the cells, and the cells were rinsed with PBS. Subsequently, a solution of 50% ethanol/49% UP water/1% glacial acetic acid was added to the wells. The absorbance of each well was read at λ_abs_ = 540 nm in a plate reader (Synergy H1, BioTek). The data were normalized as follows:

(1) $$\mathrm{Relative}\;\mathrm{Cell}\;\mathrm{Viability}=\;\frac{OD540_{treated}-OD540_{dead}}{OD540_{untreated}}$$

OD540_treated_ represents the optical density of treated cells at 540 nm, OD540_dead_ represents the optical density of dead control cells killed with 0.2 mg mL^−1^ sodium lauryl sulfate at 540 nm, and OD540_untreated_ represents the optical density of live control cells treated with MMNP@PEG-free media at 540 nm. The OD540 of live cells treated with MMNP@PEG without neutral red was negligible following the PBS rinse step, obviating the need to correct OD540 of neutral red-treated cells further.

#### 2.4.2. MMNP@PEG@RA123 Uptake Study

NIH/3T3 fibroblasts were seeded onto 35 mm tissue culture dishes (FluoroDish, World Precision Instruments, Sarasota, FL, USA) and incubated for 24 h with 0.2 µm filtered 20 µg mL^−1^ MMNP@PEG@RA123 in DMEM supplemented with 10% fetal bovine serum (FBS, Life Technologies) and 1% penicillin/streptomycin. MMNP@PEG@RA123 were prepared by post-functionalization of MMNP@PEG with RA123 in water. The cells were then rinsed with PBS and treated with Hoechst nuclear stain (Life Technologies). Standard and *z*-stack images of the live cells were taken using a Zeiss LSM 510 inverted confocal microscope (Carl Zeiss AG, Oberkochen, Germany). Hoechst staining was observed at λ_em_ = 410 nm and two-photon excitation (λ_ex_ = 760 nm), and MMNP@PEG@RA123 were visualized using λ_em_ = 525 nm and λ_ex_ = 488 nm. *z*-Stack images were taken with slices spaced evenly over 8–15 µm *z-*stack heights. Images were processed using ZEN 2.3 Lite software (Blue edition, Carl Zeiss Microscopy GmbH, Munich, Germany).

### 2.5. Statistical Analysis

Statistical analysis was performed in Minitab 17 (Minitab Inc., State College, PA, USA) by conducting analysis of variance (ANOVA) followed by post-hoc Tukey tests. Error bars in figures represent standard deviations (SD) or standard errors as specified.

## 3. Results and Discussion

### 3.1. MMNP Formation via Dopamine Autoxidation, Comparisons with Melanin, and Characterization of Surface Charge

**Figure 1:**
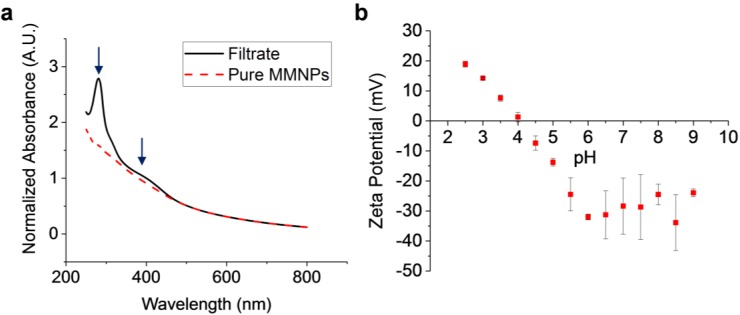
Ultraviolet–visible (UV–Vis) absorbance and surface charge of polydopamine-based melanin-mimetic nanoparticles (MMNPs). (**a**) UV-Vis spectra of purified MMNPs and filtrate removed from crude product via 10 kDa centrifugal filtration. Arrows in (**a**) indicate the two peaks observed at 280 and 398 nm in the filtrate absorbance spectrum that are absent in the purified MMNP absorbance spectrum. A.U.: Arbitrary units. (**b**) Zeta potential of MMNPs at pH 2.5–9.0. The isoelectric point is approximately pH 4.0–4.1.

After adding DA to aqueous NaOH, the solution gradually changed from colorless to yellow to dark brown-black within one hour. The purified MMNP suspension was black and demonstrated a smooth, monotonically decaying broadband UV–Vis absorbance like melanin, with highest absorbance in the UV region (Figure 1a). In contrast, measurements of the supernatant solution removed during growth show absorbance peaks at 280 and 398 nm superimposed upon this monotonically decaying curve. These peaks have been attributed to the (precursor) DA monomer and its oxidation product dopamine *o*-quinone, respectively. Since no oxidant was added to the growth solution except for ambient dissolved oxygen, the presence of both DA and dopamine *o*-quinone in the solution phase confirm that MMNP formation follows an autoxidation route [34,35]. The monotonically decaying UV–Vis absorbance in both the filtrate and pure MMNP spectra are consistent with the formation of polydopamine, some of which may be present as pre-formed oligomers below 10 kDa in the raw product (ibid.).

For each synthetic condition, MMNP extinction coefficients were calculated at wavelengths from 300 to 1000 nm (Supplementary Figure S1). The fit of these coefficients to a single exponential decay function of wavelength was excellent (r^2^ > 0.998; Supplementary Figure S1 and Table S1). Notably, these extinction coefficients match closely with values reported by Sarna et al. for melanin, especially for MMNPs with D_h_ < 50 nm (Supplementary Figure S1) [36,37]. These calculated extinction coefficients enabled rapid quantification of MMNP concentrations in our study and could be used in future work. Finally, we investigated the surface charge of MMNPs as a function of pH between pH 2.5 and 9.0 (Figure 1b) to determine the potential role of electrostatic interactions in MMNP surface loading (see Section 3.3, Section 3.4 and Section 3.5). An isoelectric point of approximately pH 4.0–4.1 was observed, which is in agreement with previous findings on polydopamine films [38]. X-ray photoelectron spectroscopy data also shows a carbon to oxygen ratio of the MMNPs that is essentially identical with polydopamine (see Section 3.3).

The chemical similarity of the MMNPs and polydopamine coatings indicates that common methods of polydopamine functionalization may be applied to the MMNPs. Moreover, the clear trend in surface ionization of MMNPs with pH suggests that electrostatic attraction may be utilized for MMNP modification in a pH-dependent manner (see Section 3.5).

### 3.2. MMNP Size Control

**Figure 2:**
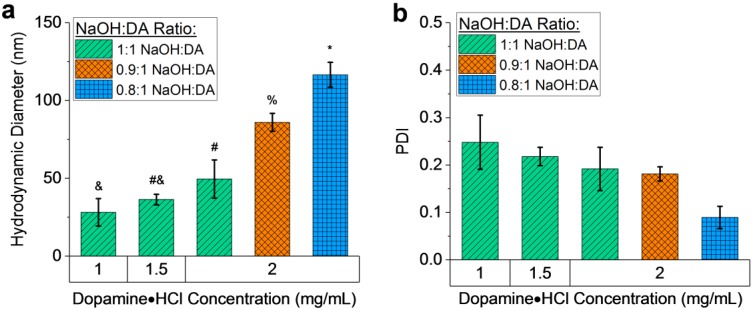
Dynamic light scattering (DLS) analysis of MMNPs. (**a**) Mean hydrodynamic diameters and (**b**) polydispersity indices (PDI) of multiple batches of MMNPs prepared at various dopamine·HCl (DA) concentrations and NaOH:DA ratios. *n* = 3−19 independently prepared batches of MMNPs were analyzed for each synthetic condition. Error bars represent standard deviations. Bars not sharing symbols in (**a**) differ significantly with *p* < 0.001.

By varying the DA concentration (1 to 4 mg mL^−1^) and NaOH:DA molar ratio (0.5:1 to 1:1) in the synthesis of MMNPs, nanoparticle D_h_ could be adjusted from 28 to 117 nm. Figure 2 shows the diameters of MMNPs as measured by DLS. Highly reproducible results were obtained by our synthetic methodology. All reported results represent the average of at least three (and up to 19) independent sample preparations.

Note that the commercially available DA is an HCl salt, and the NaOH serves to neutralize this salt as well as to increase the pH to facilitate polydopamine formation. Second, increasing DA concentration at constant NaOH:DA molar ratio resulted in larger NPs (Figure 2a and Supplementary Figure S2). Holding the NaOH:DA ratio constant at 1:1, increasing DA from 1 to 2 mg mL^−1^ resulted in an increase of D_h_ from 28.1 ± 8.8 nm to 49.5 ± 12.3 nm (mean ± SD). We attribute this to the increased quantity of dopamine available to bind to each nucleated NP. These trends demonstrate significantly finer control of NP diameter over the sub-100 nm scale compared to previous attempts to control MMNP sizes [13].

**Figure 3:**
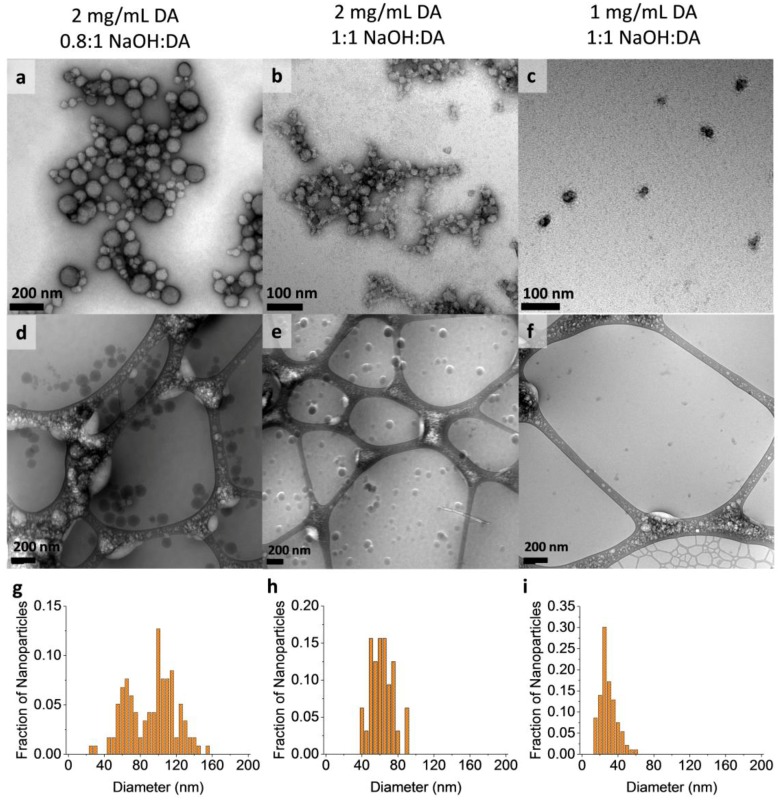
Transmission electron microscopy (TEM) images of MMNPs and quantitative analysis of nanoparticle diameter grown at the conditions specified at the top of each column. (**a**–**c**) TEM images with uranyl acetate negative stain. (**d**–**f**) Cryo-TEM images were taken without staining. Nanoparticles are spherical but have rougher appearances as diameter decreases. (**g**–**i**) Distribution of MMNP diameters in cryo-TEM images.

Although the average D_h_ values were highly consistent from batch to batch, this consistency must be distinguished from the variance in MMNP diameter within each batch, which was assessed initially by PDI. The average PDI of NP batches prepared at each condition ranged from 0.09 to 0.25 (Figure 2b). This result indicates that individual MMNP batches are relatively monodisperse for organic NPs but that size analysis beyond cumulants analysis of DLS data is required [39]. As such, the polydispersity and morphology of MMNPs were further assessed by SEM, TEM, and cryo-TEM (Figure 3; see also Supplementary Figures S3 and S4, and Table S2). Spherical NPs were always observed. The imaging data also corroborate the DLS data demonstrating that NP size increased as DA concentration increased and as NaOH:DA ratio decreased (Figure 3).

Unimodal size distributions were observed for synthesis conditions that produced MMNPs up to a diameter of ≈50 to 60 nm (Figure 3). Minimal MMNP aggregation was observed in images obtained by cryo-TEM, which does not suffer from the drying artifacts of conventional TEM and SEM, confirming that the products mainly consisted of dispersed NPs. In particular, low polydispersities were obtained for MMNPs produced both at 1 mg mL^−1^ and 2 mg mL^−1^ DA with 1:1 NaOH:DA (SD of 9.1 nm and 12.4 nm were observed, respectively; Figure 3h,i and Supplementary Table S2). However, the NP size distribution was bimodal at 2 mg mL^−1^ DA with only 0.8:1 NaOH (Figure 3g), with distinct NP populations centering around D_h_ = 65 nm and 100 nm. Because the DLS signal intensity is related to the 6th power of the particle diameter (i.e., weighted more heavily toward the larger nanoparticles), this cryo-TEM result is consistent with the DLS data shown in Figure 2 indicating an average D_h_ = 120 nm at this condition.

### 3.3. PEGylation to Produce MMNP@PEG

We focused on the 49.5 nm MMNPs for PEGylation studies because these NPs would remain within a biologically useful size regime following modification (i.e., D_h_ <100 nm). PEGylation was achieved by overnight treatment with 10 mM 5 kDa mPEG-SH in 10 mM NaOH, and the NPs were evaluated using zeta potential, DLS, and XPS data. Control batches of MMNPs were treated with 10 mM NaOH base without mPEG-SH.

**Figure 4:**
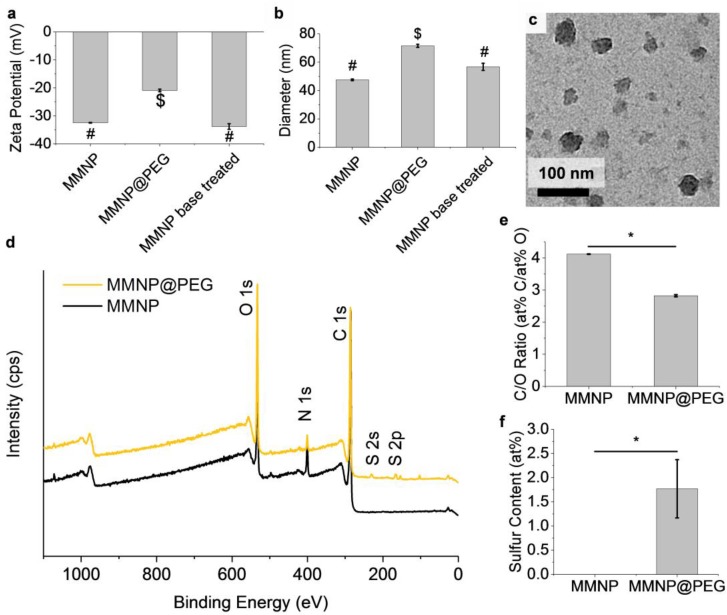
MMNP@PEG vs. MMNP zeta potential, hydrodynamic diameter, morphology, and atomic composition. (**a**) Zeta potentials and (**b**) hydrodynamic diameters of MMNPs, MMNP@PEG, and control MMNPs treated with 10 mM NaOH base. Samples not sharing symbols are significantly different (*p* < 0.05). (**c**) TEM image of MMNP@PEG. (**d**) XPS survey scans of MMNP and MMNP@PEG with assignments for O 1s, N 1s, C 1s, and S 2s, and S 2p peaks. (**e**) C/O atomic ratios in MMNP vs. MMNP@PEG calculated from C 1s and O 1s signal ratios (* *p* < 0.01). at%: Atomic percent relative to total C, N, O, and S content. (**f**) Sulfur content in MMNP vs. MMNP@PEG calculated from S 2p signal intensity expressed as at% S (* *p* < 0.01). Error bars represent standard errors.

The zeta potential of the resulting MMNP@PEG was −20.9 ± 0.5 mV at pH 7.4, which was significantly higher than those of both untreated MMNPs (−32.5 ± 0.1 mV) and base treated MMNPs (-33.8 ± 1.0 mV) at pH 7.4 (Figure 4a), indicating shielding of the negatively charged polydopamine MMNP surface. Consistent with the zeta potential results, DLS shows PEGylation increased the D_h_ of the MMNPs by 24 nm to 71.5 ± 1.1 nm (Figure 4b). Melanin-mimetic nanoparticles treated with base alone did not have a significantly greater D_h_ than untreated MMNPs, confirming that the NP diameter increase was not caused by MMNP growth or aggregation in basic conditions. Additionally, TEM imaging shows that MMNP@PEG have spherical morphology similar to that of MMNPs (Figure 4c), validating the use of the standard spherical NP analysis of the DLS data.

The thickness of the PEG layer is 12 nm (half of the change in D_h_), twice the thickness that would be expected from the mushroom regime [40,41], providing evidence that PEG is packed in the brush regime rather than the mushroom regime at the surface of MMNPs. A PEG brush causing a similar diameter increase has also been reported previously to provide sufficient resistance against protein adsorption and phagocytosis on other organic and inorganic NP cores, including poly(lactic acid) (PLA), poly(lactic-co-glycolic acid) (PLGA), polycaprolactone (PCL), and gold [42,43,44,45,46,47].

PEGylation was further corroborated by XPS analysis of drop-casted NPs suspensions (Figure 4d). In addition to O 1s, N 1s, and C 1s signals, the survey spectrum of MMNP@PEG reveals the presence S 2s and S 2p signals, indicating the presence of sulfur from mPEG-SH. Sulfur peaks were absent in unmodified MMNP controls. Moreover, the C/O atomic ratios decreased from 4.12 ± 0.01 for unfunctionalized MMNP controls to 2.82 ± 0.03 for MMNP@PEG. Since MMNP@PEG is compositionally a mixture of polydopamine and PEG, this latter C/O ratio is consistent with successful PEGylation because it is intermediate between the theoretical ratio of 4 for dopamine and its oxidation products and a ratio of 2 for mPEG-SH. Furthermore, while high resolution C 1s and O 1s spectra for MMNPs show π–π*, C=O, C–O/C–N, C–H_x_/C–C chemical shifts corresponding to previously reported polydopamine coatings (Supplementary Figure S5) [48,49], the spectra of MMNP@PEG show large increases of C–O components, demonstrating the presence of PEG on the MMNP@PEG.

### 3.4. Stability of MMNP@PEG

The stabilities of MMNP and MMNP@PEG samples were compared by immersion in 1× PBS and in cell culture media (DMEM + 10% serum) for 24 h. Before PEGylation, MMNPs were stable in UP water but aggregated in 1× PBS (Supplementary Figure S6a). Thus, the electrostatic repulsion between unfunctionalized MMNPs was insufficient in maintaining colloidal stability with screening at physiologic ionic strength. Incidentally, in cell culture media with serum, MMNPs do not visibly aggregate and appeared to be stable (Supplementary Figure S6b). Potentially, this effect is due to the sterically stabilizing effect of serum proteins bound to the polydopamine surface of MMNPs, as it is known that amine groups in proteins can covalently bind to the polydopamine surfaces at physiologic pH [50]. In contrast, MMNP@PEG remained stable for 24 h in both 1× PBS and cell culture media (Supplementary Figure S6c,d). Even after fluorophore modification, it was noted that MMNP@PEG remained stable in 1× PBS for up to seven days (see Section 3.5.1 and Section 3.5.2).

### 3.5. Fluorescence Functionalization

**Figure 5:**
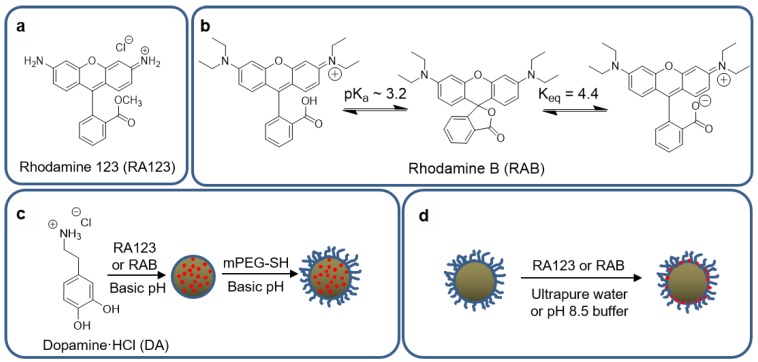
Approaches to synthesis of fluorescent MMNPs. (**a**) Structure of rhodamine 123 (RA123). (**b**) Structures of rhodamine B (RAB), including the fluorescent cationic acid, non-fluorescent neutral lactone, and fluorescent zwitterionic structures. (**c**) In situ approach and subsequent PEGylation: MMNP@RA123 and MMNP@RAB are first synthesized by DA polymerization in the presence of RA123 or RAB. These fluorescent NPs are subsequently PEGylated, forming MMNP@RA123@PEG and MMNP@RAB@PEG. (**d**) Post-functionalization approach: MMNP@PEG@RA123 and MMNP@PEG@RAB are formed by treatment of MMNP@PEG with RA123 or RAB in unbuffered ultrapure water or pH 8.5 buffer.

We compared two methods of MMNP functionalization with small organic fluorophores (Figure 5): an in situ method in which the fluorescent molecules were mixed and incorporated with dopamine during MMNP formation, and a post-functionalization method in which the fluorophores were added onto purified MMNP@PEG. Both RA123 and RAB were used as model fluorophores. Both may interact with polydopamine via π–π stacking, hydrogen bonding, or electrostatic interactions. In addition, RA123 has a primary amine that may behave as a weak nucleophile to covalently bind to oxidized quinones in polydopamine (Figure 5a). The predominant structure of RAB is the fluorescent zwitterion, but a significant fraction of RAB also exists as a non-fluorescent lactone, with K_eq_ = [zwitterion]/[lactone] = 4.4 at 25 °C in water (Figure 5b) [51]. The acidic cation of RAB has pK_a_ ≈ 3.2 but has been reported to increase up to 5.70 in the presence of microheterogeneities in solution, such as the interfaces formed by surfactants, thus stabilizing it at higher pH than in homogeneous solutions [52]. These molecules and their modes of binding may also be viewed as models for the incorporation of other functionalities, such as chemotherapeutics.

#### 3.5.1. In situ Incorporation

Melanin-mimetic nanoparticles were labeled in situ by growing MMNPs in 1 mg mL^−1^ DA and 1:1 NaOH:DA in the presence of 50 µg mL^−1^ RA123 or RAB to prepare MMNP@RA123 or MMNP@RAB, respectively (Figure 5c). These NPs were then modified with 5 kDa mPEG-SH to form MMNP@RA123@PEG and MMNP@RAB@PEG. In order to remove loosely bound dye, samples were centrifugally filtered with extensive washing and then dialyzed for seven days in 1× PBS. Approximately 90% of the physisorbed dye remaining after centrifugal filtration was released within the first 24 h of dialysis (Supplementary Figure S7a). No aggregation was observed during immersion in PBS for one week, indicating good steric stability imparted by the PEG coating.

**Figure 6:**
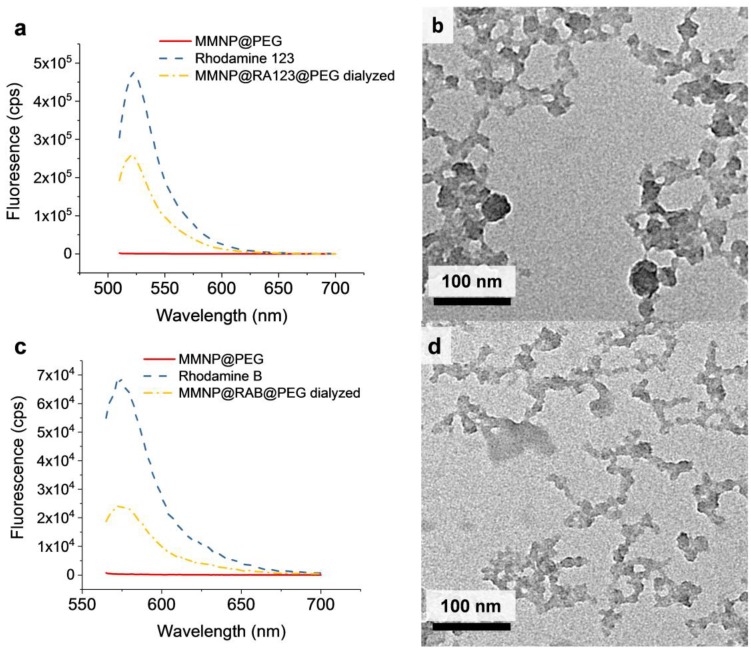
Fluorescence emission spectra and TEM images of in situ labeled MMNP@RA123 and MMNP@RAB. (**a**) Fluorescent emission spectra (λ_ex_ = 500 nm) of MMNP@RA123@PEG after seven day dialysis in 1× phosphate-buffered saline (PBS), rhodamine 123, and MMNP@PEG. (**b**) TEM image of MMNP@RAB. (**c**) Fluorescent emission spectra (λ_ex_ = 555 nm) of MMNP@RAB@PEG after seven day dialysis in 1× PBS, rhodamine B, and MMNP@PEG. (**d**) TEM image of MMNP@RA123.

In the first step of MMNP growth in solution mixtures of DA and rhodamine, both UV–Vis absorbance spectroscopy and fluorimetry provided evidence that RA123 and RAB were successfully incorporated into the in situ labeled NPs and retained after centrifugal filtration (Supplementary Figures S8 and S9a,b). The fluorescence emission peaks of MMNP@RA123@PEG and MMNP@RAB@PEG were centered at 520 nm and 573 nm, respectively, similar to the free dyes, and remained at those locations following extensive dialysis (Figure 6a,c). The 10 nm red-shift in the MMNP@RA123@PEG absorbance peak (λ_abs_ = 510 nm) relative to free RA123 (λ_abs_ = 500 nm) may indicate some dye aggregation in the NPs. After seven day dialysis in 1× PBS, the fluorescent signal of a 25 µg mL^−1^ solution of MMNP@RA123@PEG approximately corresponds to that of 5.4 ng mL^−1^ free RA123, and the fluorescence of a 25 µg mL^−1^ solution of MMNP@RAB@PEG approximately corresponds to that of 3.6 ng mL^−1^ free RAB. These in situ-modified NPs also have full-width half-maximum (FWHM) of approximately 45 nm, which is two to three times narrower than previously reported fluorescent polydopamine NP systems [26,27,28,30]. The broadband UV–Vis absorbance pattern also verified that polydopamine growth could proceed in the presence of rhodamine (Supplementary Figure S8). Furthermore, the largely negative zeta potentials of both MMNP@RA123 and MMNP@RAB were not significantly different from those of MMNPs without rhodamine, indicating that the dyes were chiefly incorporated into the interior of the NPs (Supplementary Figure S10a). Otherwise, the positively charged RA123 or various forms of RAB would have increased the zeta potential significantly versus vs. MMNPs by masking the negative MMNP surface charge or by reversing it, especially if the fluorophores segregated to the NP surface.

After PEGylation, zeta potential measurement provided evidence that grafting the polydopamine NP surfaces with mPEG-SH was successful, as the zeta potentials became significantly less negative (−9.9 ± 1.4 mV for MMNP@RA123@PEG and −7.6 ± 1.9 mV for MMNP@RAB@PEG, vs. −34.4 ± 0.8 mV for MMNP@RA123 and −39.3 ± 1.3 mV for MMNP@RAB; Supplementary Figure S10a). However, a high polydispersity interfered with quantitative use of DLS data (Supplementary Figure S10b), and an increase in D_h_ following PEGylation could not be confirmed. In fact, TEM shows that these NPs were more polydisperse and less well-defined than MMNPs grown without dye (Figure 6b,d). It is possible that a lower level of rhodamine incorporation could restore normal MMNP growth and this could be worth pursuing in future work given the encouraging fluorescence profile, colloidal stability, and straightforward synthesis of the in situ modified NPs.

#### 3.5.2. Post-Functionalization

MMNP@PEG (40 µg mL^−1^; D_h_ = 71.5 ± 0.6 nm) were post-functionalized by incubating in 50 µg mL^−1^ RA123 or RAB dye to form MMNP@PEG@RA123 or MMNP@PEG@RAB, respectively (Figure 5d). Two solution conditions were tested: functionalization in UP water and in buffer at pH 8.5. Both RA123 and RAB may modify the free polydopamine surface remaining in between the PEG chains via non-covalent interactions such as π–π stacking or hydrogen bonding. The positive charge of RA123 could also promote more electrostatic attraction to the negatively charged polydopamine surface than RAB. The primary amine on the RA123 could undergo Michael addition for covalent binding to polydopamine especially at the pH 8.5 basic condition as well [50], although this coupling may not be prominent, since the aromatic primary amine is a weak nucleophile [53].

**Figure 7:**
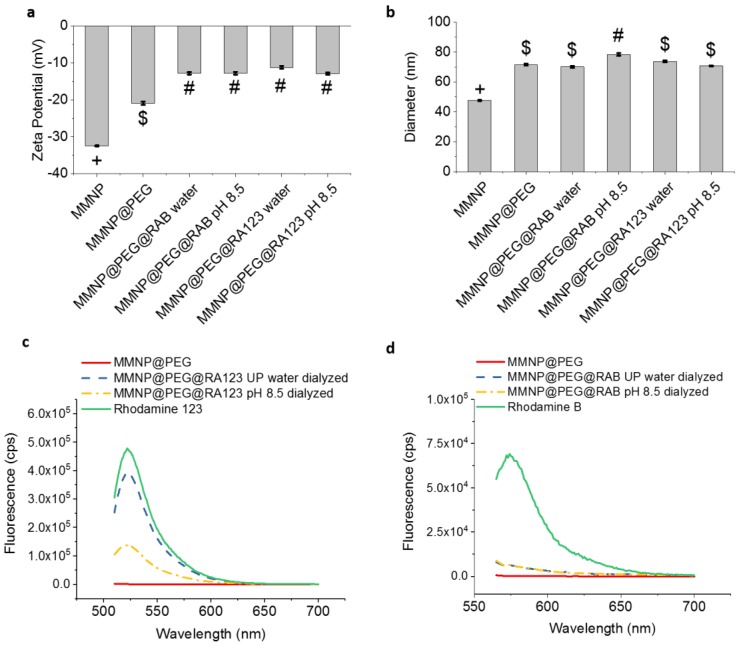
Fluorescence, hydrodynamic diameter, and zeta potential of rhodamine post-functionalized MMNP@PEG. (**a**) Zeta potentials and (**b**) hydrodynamic diameters of rhodamine post-functionalized MMNP@PEG samples prepared in water or at pH 8.5 vs. unmodified MMNPs and MMNP@PEG. Groups not sharing symbols have significantly different values (*p* < 0.05). (**c**) Fluorescence emission spectra (λ_ex_ = 500 nm) of 25 µg mL^−1^ samples of MMNP@PEG before and after modification with RA123 in water or at pH 8.5 followed by serial dialysis in ultrapure (UP) water for 72 h and 1× PBS for 72 h. Emission spectrum of RA123 was taken at 10 ng mL^−1^. (**d**) Fluorescence emission spectra (λ_ex_ = 555 nm) of 25 µg mL^−1^ samples of MMNP@PEG before and after modification with RAB in water or at pH 8.5 followed by serial dialysis in UP water for 72 h and 1× PBS for 72 h. Emission spectrum of RAB was taken at 10 ng mL^−1^.

Rhodamine functionalization was first confirmed by the appearance of prominent absorption peaks in UV–Vis spectra and fluorescence emission spectra taken directly following extensive centrifugal filtration to remove the dissolved free dye in the solution used for functionalization (Supplementary Figures S9c,d and S11). The red-shifted absorbance peaks on MMNP@PEG@RA123 (**λ**_abs_ = 520 nm) vs. free RA123 (**λ**_abs_ = 500 nm) indicate that the RA123 has aggregated on the NP surface, potentially due to high loading. No obvious differences were noted between samples modified in UP water or at pH 8.5. Moreover, both MMNP@PEG@RAB and MMNP@PEG@RA123 have significantly higher zeta potentials than MMNP@PEG (Figure 7a), further indicating coverage of and binding to the polydopamine NP surface underlying the PEG brush. The finding that the zeta potentials for all of the post-functionalized NPs were similar may indicate that the cationic form of RAB is stabilized at the negatively charged MMNP surface, as observed in microheterogeneous solutions containing surfactant micelles [52]. Dynamic light scattering measurements show that the NP diameter generally did not increase after dye functionalization, except for a <10% increase for MMNP@PEG@RAB modified at pH 8.5 (Figure 7b). It is thus unlikely that polydopamine growth or NP aggregation occurred during fluorophore loading.

To ensure that the fluorescent emission of MMNP@PEG@RA123 and MMNP@PEG@RAB was due to the fluorophores bound to the MMNPs and that this emission would be stable, additional dialysis was performed after centrifugal filtration—72 h in UP water followed by a further 72 h in 1× PBS—to remove dye molecules that could be desorbed from the MMNPs. The dialysis process was successful in removing this loosely bound fraction (over 80% of removable fraction of dyes was released within the first 24 h of the first UP water dialysis) (Supplementary Figure S7b,c). Although measurements of the emission levels during dialysis do show that a large portion of the initially measured fluorescence was due to loosely bound dyes that desorb from the NP surface (Supplementary Figure S7b,c), the fluorescence of MMNP@PEG@RA123 was still detectable (Figure 7c). The emission peaks of MMNP@PEG@RA123 centered at λ_em_ = 520–524 nm, which are essentially unchanged from the free dye. The fluorescence remaining in 25 µg mL^−1^ samples of MMNP@PEG@RA123 corresponded to 2.9 ng mL^−1^ RA123 for the pH 8.5 modification condition and 8.3 ng mL^−1^ RA123 for the UP water modification condition. Thus, the RA123 remained strongly bound to the MMNP@PEG@RA123 surface, and the pH 8.5 condition did not enhance interactions between aromatic amines on RA123 and polydopamine vs. UP water.

On the other hand, no emission peaks are observed in dialyzed MMNP@PEG@RAB samples (Figure 7d). Taken together with zeta potential results, which suggest the presence of the acidic cation of RAB at the MMNP surface, the almost total removal of RAB after dialysis also suggests an electrostatic binding mechanism: During dialysis, the bound fluorescent RAB cation may equilibrate with the non-fluorescent lactone and fluorescent zwitterionic RAB forms, which may subsequently desorb from the NP surface due to less electrostatic attraction to polydopamine.

We also observed that during the dialysis process, more RA123 was released in the first UP water dialysis step for samples prepared at pH 8.5 than in UP water. This result indicates that covalent bonding is not preferred at pH 8.5 and that electrostatic attraction between RA123 and polydopamine may be the preferred mechanism of RA123 loading onto the MMNP surface. Regardless, the fluorescence of the MMNP@PEG@RA123 after extensive dialysis also shows that this physical binding is sufficient to obtain stable fluorescent NPs. More RA123 was released from samples prepared in UP water in the second dialysis step in 1× PBS. The origin of this effect is unclear. Nevertheless, the level of RA123 fluorescence retained on NPs functionalized in UP water was significantly higher, and this approach was used to generate MMNP@PEG@RA123 for cell work.

### 3.6. In Vitro Cytocompatibility of MMNP@PEG and Imaging of MMNP@PEG@RA123

**Figure 8:**
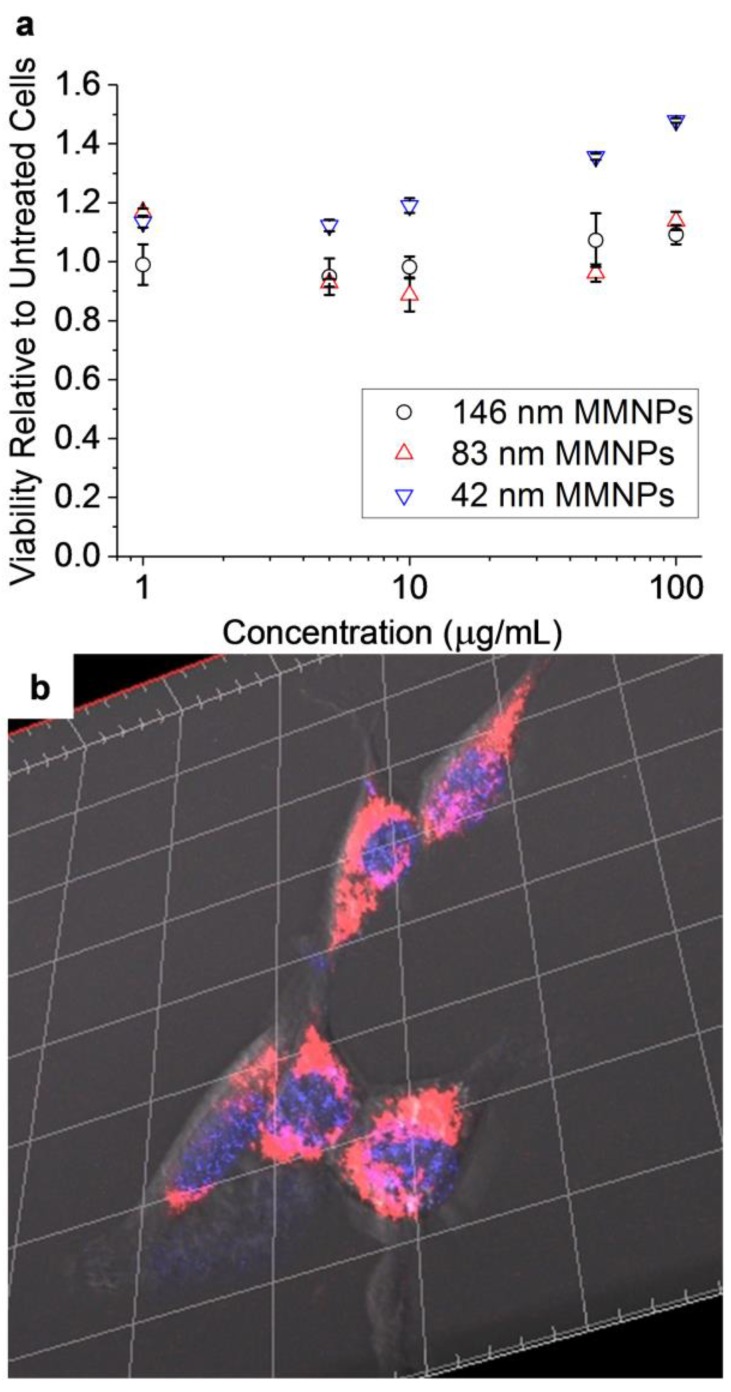
In vitro investigation of MMNP-cell interactions. (**a**) MMNP cytocompatibility with NIH/3T3 fibroblasts as measured by neutral red uptake viability assay. Error bars represent standard errors of triplicate experiments. (**b**) Representative confocal microscopy three-dimensional (3D) *z*-stack reconstruction image of Hoechst-stained NIH/3T3 fibroblasts treated with 20 µg mL^−1^ MMNP@PEG@RA123. Hoechst stain (blue) and rhodamine fluorescence (red/pink) are shown here; Scale bar: 20 µm between gridlines.

The viability of NIH/3T3 fibroblasts incubated in media loaded with MMNP@PEG was evaluated. A range of NPs with D_h_ = 42 nm to 146 nm were tested (the diameters refer to the values measured for the specific batch of NPs used for each viability assay rather than the averages shown in Figure 2). No toxicity was observed over a duration of 24 h at all tested concentrations (1–100 µg mL^−1^; Figure 8a). In fact, some increase in relative cell viability was observed for cells treated with MMNP@PEGs, most notably for the smallest 42 nm diameter tested (up to 40% higher). This effect was previously observed in HeLa cells at 6–75 µg mL^−1^ treatments, but not in 4T1 cells [13,23]. It is possible that this dose-dependent effect stems from the known antioxidant capacity of MMNPs [13], which may alter cellular proliferation by limiting oxidative stress in some cells. Finally, confocal microscopy was used to characterize the cell uptake of MMNP@PEG@RA123. Figure 8b shows a representative three-dimensional (3D) *z*-stack composite reconstruction of the NIH/3T3 fibroblasts treated with both Hoechst dye and MMNP@PEG@RA123 (separately imaged with 760 nm two-photon and regular 488 nm excitation, respectively). After 24 h incubation, the fluorescence associated with the MMNP@PEG@RA123 could be clearly observed, even at the relatively low incubation concentration of 20 µg mL^−1^. Additional *z*-stack confocal images and 3D reconstructions of treated cells vs. untreated control cells confirmed that the observed fluorescence was located within the cells, indicating MMNP internalization (Figure 8b and Supplementary Figures S12–S16). Co-staining the cells with the Hoechst dye used for nuclear staining revealed that the MMNP@PEG@RA123 was concentrated in the perinuclear region—they were excluded from both the cell nuclei and the filapodia regions. It was also observed that MMNP@PEG@RA123 have a punctate distribution within cells. From these confocal microscopy images, it is evident that MMNP@PEG@RA123 is sufficiently stable to be utilized in high-resolution, multimodal cell imaging.

## 4. Conclusions

We have demonstrated spherical MMNPs labeled with fluorescent dyes with controlled diameters. Careful measurements based on multiple independent batches of NP preparation (up to 19) showed how adjustment of DA concentration and NaOH:DA ratio in MMNP synthesis could be used to achieve fine control of batch mean MMNP diameter in the sub-100 nm range. Similarly, our conditions for MMNP PEGylation produced particles with a high degree of stability in both 1× PBS and in cell culture media. Melanin-mimetic nanoparticle formation was shown to follow an autoxidation route, and the similarities of MMNPs with polydopamine and melanin in terms of chemical identity and surface charge were shown by UV–Vis absorption, zeta potential, and XPS analysis. No cytotoxicity was observed over the entire range of diameters from ≈40 to 150 nm tested.

We also compared two approaches of loading MMNPs with aromatic fluorescent dyes—in situ dye loading during MMNP formation and post-functionalization after MMNP formation and PEGylation. The fluorescence spectra of MMNPs obtained using both protocols produced emission peak widths ≈40 nm FWHM, similar to the free dye and less than half that of previous reports of fluorescent polydopamine NPs. The in situ approach, however, modified the shape of the MMNPs, but post-functionalization could be used to produce spherical MMNPs with stable fluorescence suitable for high-resolution multimodal confocal live cell imaging.

The reproducible diameter control and facile methodologies for functionalizing and loading the MMNPs are highly applicable to fundamental studies and further refinement of organic NP–cell interactions, such as size-dependent cellular uptake and intracellular trafficking using targeting moieties. The stable and spectrally narrow fluorescence measured indicate that our protocol could be beneficial for incorporating dyes into MMNPs (or other polydopamine matrices) for multimodal imaging studies, or for delivery of therapeutic molecules with weak interactions to MMNPs.

## Supplementary Materials

The following are available online at http://www.mdpi.com/2313-7673/2/3/17/s1: Figure S1: Extinction coefficients of melanin and MMNPs prepared at various synthetic conditions, Figure S2: Melanin-mimetic nanoparticle diameter dependence on [NaOH]:[DA] and DA concentration, Figure S3: Scanning electron microscopy image of MMNPs prepared in 2 mg mL^−1^ DA with 1:1 NaOH:DA, Figure S4: Transmission electron microscopy characterization of MMNPs, Figure S5: Melanin-mimetic nanoparticle and MMNP@PEG high-resolution C 1s and O 1s XPS peaks with peak deconvolutions, Figure S6: Evaluation of MMNP and MMNP@PEG stability in 1× PBS and DMEM + 10% serum, Figure S7: Dialysis of rhodamine-labeled MMNPs in water and PBS following synthesis and centrifugal filtration, Figure S8: Ultraviolet–visible absorbance spectra of in situ labeled MMNPs, Figure S9: Fluorescence emission spectra of rhodamine 123- and rhodamine B-labeled MMNPs following centrifugal filtration and before dialysis, Figure S10: Zeta potential and dynamic light scattering characterization of in situ labeled MMNPs, Figure S11: Ultraviolet–visible absorbance spectra of rhodamine-treated MMNP@PEG, Figure S12: Confocal *z*-stack images of control Hoechst-stained NIH/3T3 fibroblasts untreated with MMNP@PEG@RA123, Figure S13: Confocal *z*-stack images of Hoechst-stained NIH/3T3 fibroblasts treated for 24 h with 20 µg mL^−1^ MMNP@PEG@RA123, Figure S14: Three-dimensional reconstruction of confocal *z*-stack images of control Hoechst-stained NIH/3T3 fibroblasts untreated with MMNP@PEG@RA123, Figure S15: Three-dimensional reconstructions of confocal *z*-stack images of Hoechst-stained NIH/3T3 fibroblasts treated for 24 h with 20 µg mL^−1^ MMNP@PEG@RA123, Figure S16: A second area covered by three-dimensional reconstruction of confocal *z*-stack images of Hoechst-stained NIH/3T3 fibroblasts treated for 24 h with 20 µg mL^−1^ MMNP@PEG@RA123, Table S1: Parameters for exponential decay fitting of extinction coefficient vs. wavelength in Supplementary Figure S1, Table S2: Comparison of MMNP size distributions calculated by DLS and cryo-TEM for the samples analyzed in Figure 3d–i.
