# Supplementary material for: Size Control and Fluorescence Labeling of Polydopamine Melanin-Mimetic Nanoparticles for Intracellular Imaging
Source: Biomimetics (Basel). 2017 Sep 6;2(3):17. doi: 10.3390/biomimetics2030017 (PMC5774220; doi:10.3390/biomimetics2030017)
Supplement: Supplementary file 1 [file biomimetics-02-00017-s001.pdf]

Biomimetics **2017**, *2*, 17

Supplementary Materials: Size Control and Fluorescence Labeling of Polydopamine Melanin-Mimetic Nanoparticles for Intracellular Imaging

Devang R. Amin [1,2], Caroline Sugnaux [1], King Hang Aaron Lau [3] and Phillip B. Messersmith [1,*]

1 Departments of Bioengineering and Materials Science and Engineering, University of California, Berkeley, 210 Hearst Mining Building, Berkeley, CA 94720, USA; damin@berkeley.edu (D.R.A.); csugnaux@berkeley.edu (C.S.)
2 Department of Biomedical Engineering, Northwestern University, 2145 Sheridan Rd., Evanston, IL 60208, USA
3 WestCHEM/Department of Pure and Applied Chemistry, University of Strathclyde, 295 Cathedral St., Glasgow G1 1XL, UK; aaron.lau@strath.ac.uk
* Correspondence: philm@berkeley.edu; Tel.: +1-510-643-9631

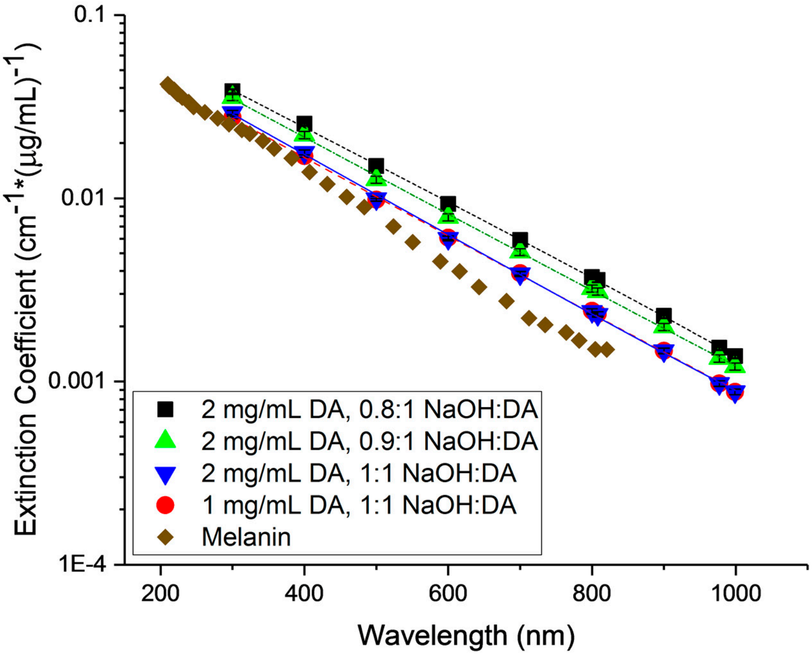

Figure S1. Extinction coefficients of melanin and of MMNPs prepared at various synthetic conditions. Exponential decay fits are shown for the MMNP extinction coefficient data. Data for melanin extinction coefficients were obtained from Sarna et al. [1,2]. Mean nanoparticle diameter of MMNPs prepared at each set of synthetic conditions are noted in the legend. Error bars are standard errors of extinction coefficients calculated from three to five independently prepared MMNP batches at each synthetic condition.

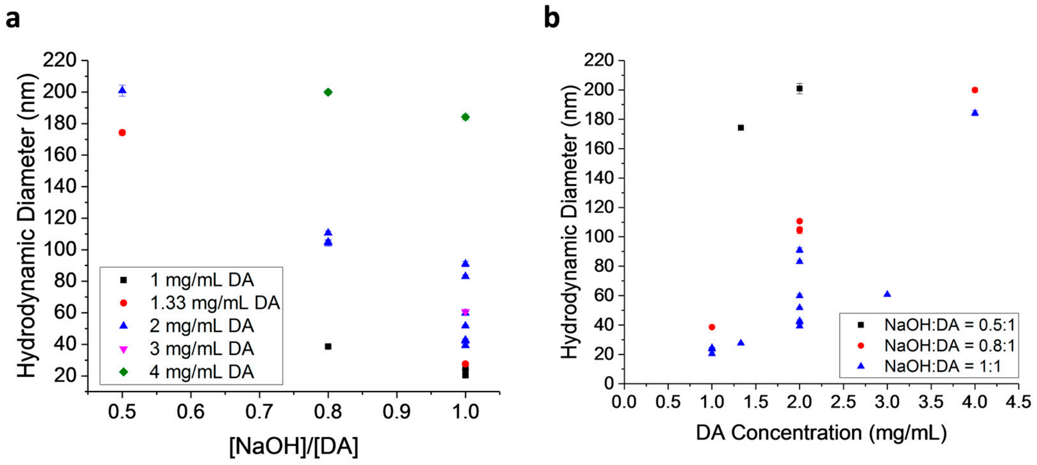

Figure S2. Melanin-mimetic nanoparticle diameter dependence on (**a**) [NaOH]/[DA] and (**b**) DA concentration during synthesis. All points represent the mean diameter of an independently prepared batch of nanoparticles as measured by DLS. Reaction time (5 h) and temperature (50 °C) were held constant for all syntheses. Nanoparticle diameter is negatively correlated with NaOH:DA at each concentration of DA used and is positively correlated with DA concentration at each NaOH:DA ratio. Error bars represent standard deviations of three DLS measurements on one batch of MMNPs.

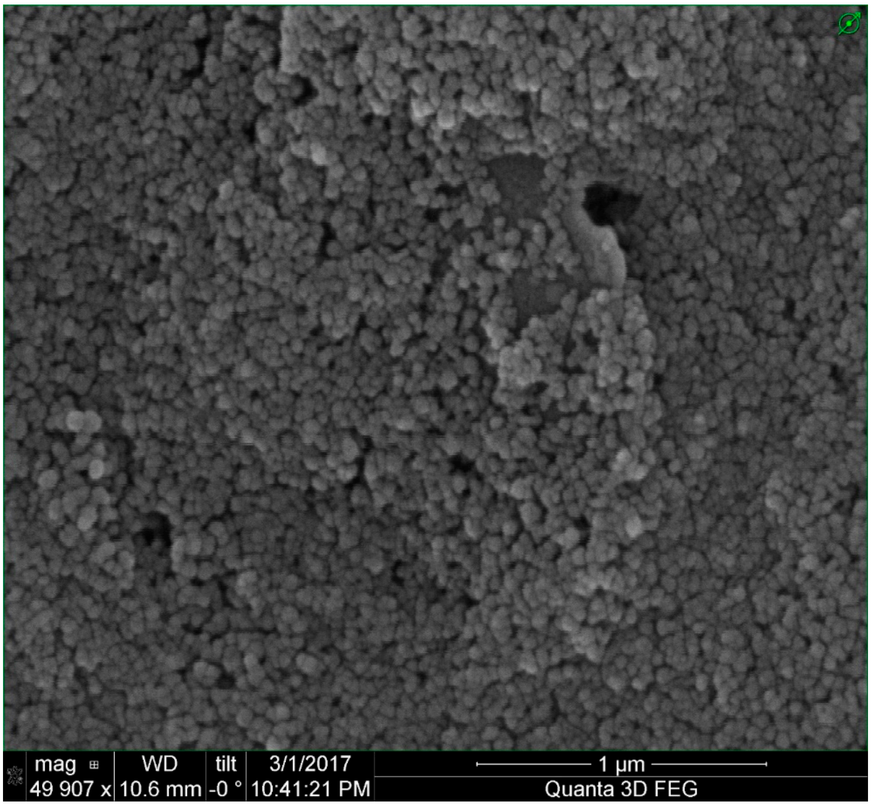

Figure S3. Scanning electron microscopy image of MMNPs prepared in 2 mg mL^{-1} DA with 1:1 NaOH:DA.

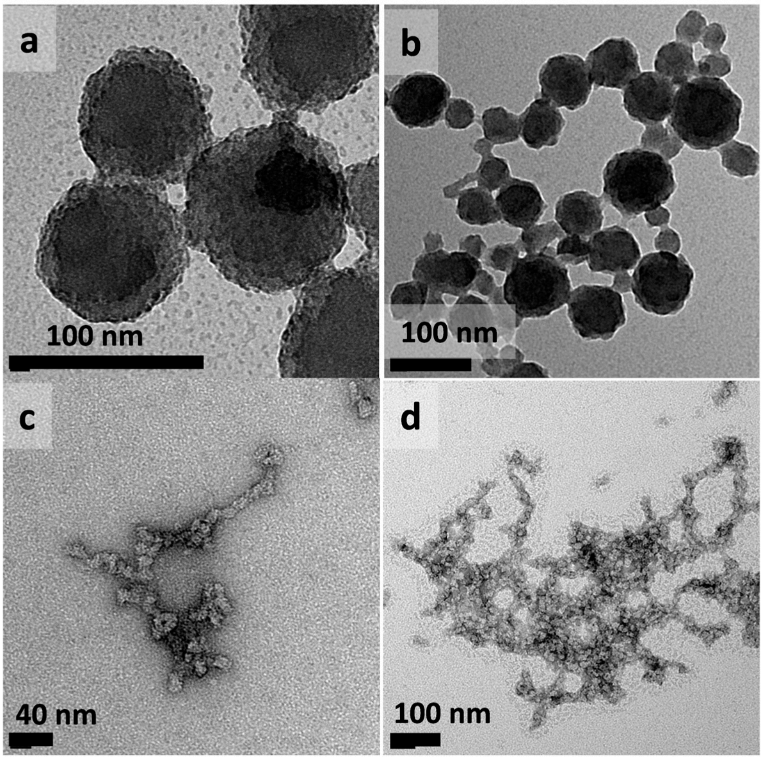

Figure S4. Transmission electron microscopy characterization of MMNPs. (**a**,**b**) TEM images of MMMPs prepared at 2 mg mL^{-1} DA with 0.8:1 NaOH:DA without negative staining. (**c**,**d**) TEM images of MMNPs prepared at 1 mg mL^{-1} DA with 1:1 NaOH:DA with negative staining.

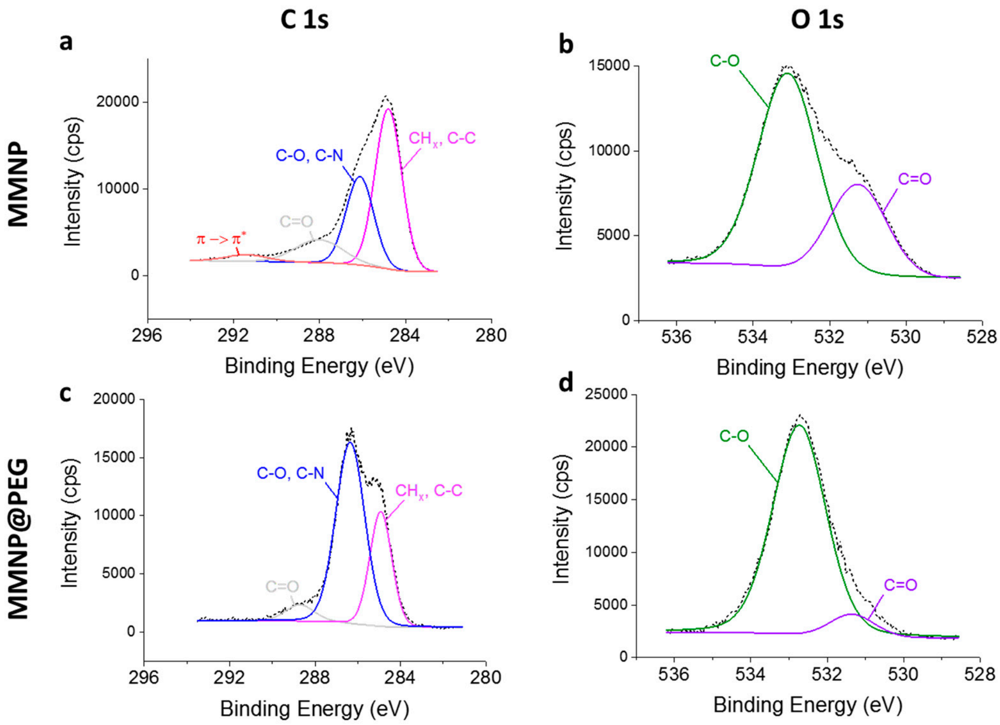

Figure S5. Melanin-mimetic nanoparticle and MMNP@PEG high-resolution C 1s and O 1s XPS peaks with peak deconvolutions. (**a**) High-resolution C 1s scan of MMNPs. (**b**) High-resolution O 1s scan of MMNP. (**c**) High-resolution C 1s scan of MMNP@PEG. (**d**) High-resolution O 1s scan of MMNP@PEG.

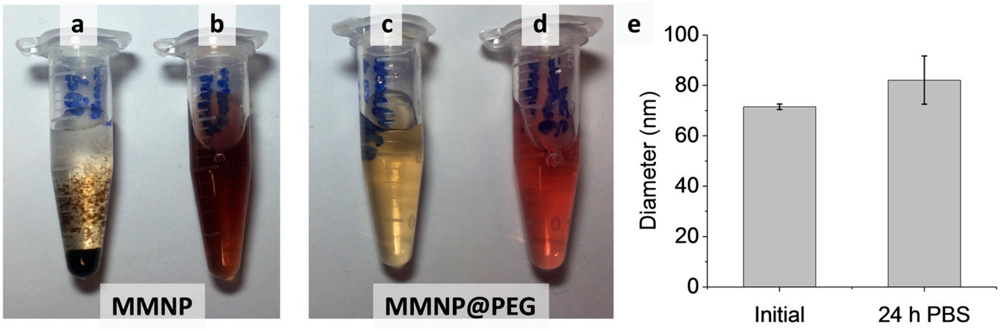

Figure S6. Evaluation of MMNP and MMNP@PEG stability in 1× PBS and DMEM + 10% serum. (**a**–**d**) Images of MMNPs and MMNP@PEG after 24 h incubation in 1× PBS or DMEM + 10% serum. After 24 h, MMNPs aggregate in 1× PBS (**a**) but not in DMEM + 10% serum (**b**). MMNP@PEG remain stable in both 1× PBS (**c**) and in DMEM + 10% serum (**d**). (**e**) Dynamic light scattering demonstrates no significant size increase of MMNP@PEG placed into 1× PBS for 24 h.

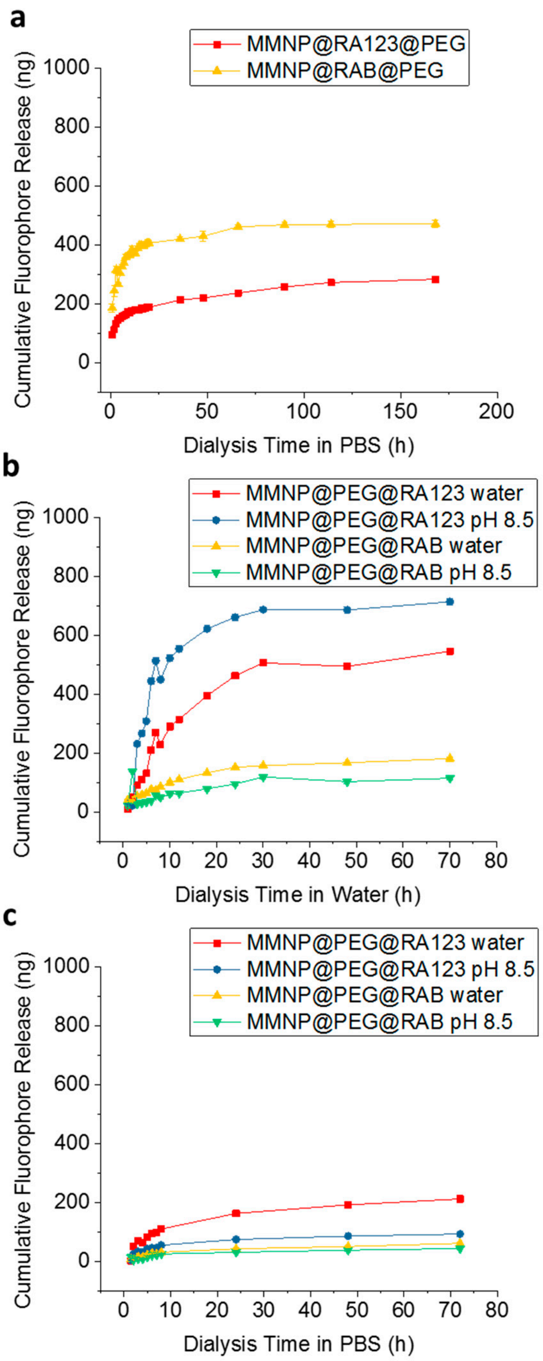

Figure S7. Dialysis of rhodamine-labeled MMNPs in water and PBS following synthesis and centrifugal filtration. (**a**) Representative rhodamine release curves during dialysis of 100 µg batches of in situ labeled MMNP@RA123@PEG and MMNP@RAB@PEG in PBS. (**b**) Representative rhodamine release curves during dialysis in water of 50 µg batches of post-functionalized MMNP@PEG@RA123 and MMNP@PEG@RAB samples prepared by modification in either water or in pH 8.5 buffer. (**c**) After dialysis for 72 h in water, the samples from (**b**) were placed into PBS, and release of rhodamine dyes was tracked in PBS.

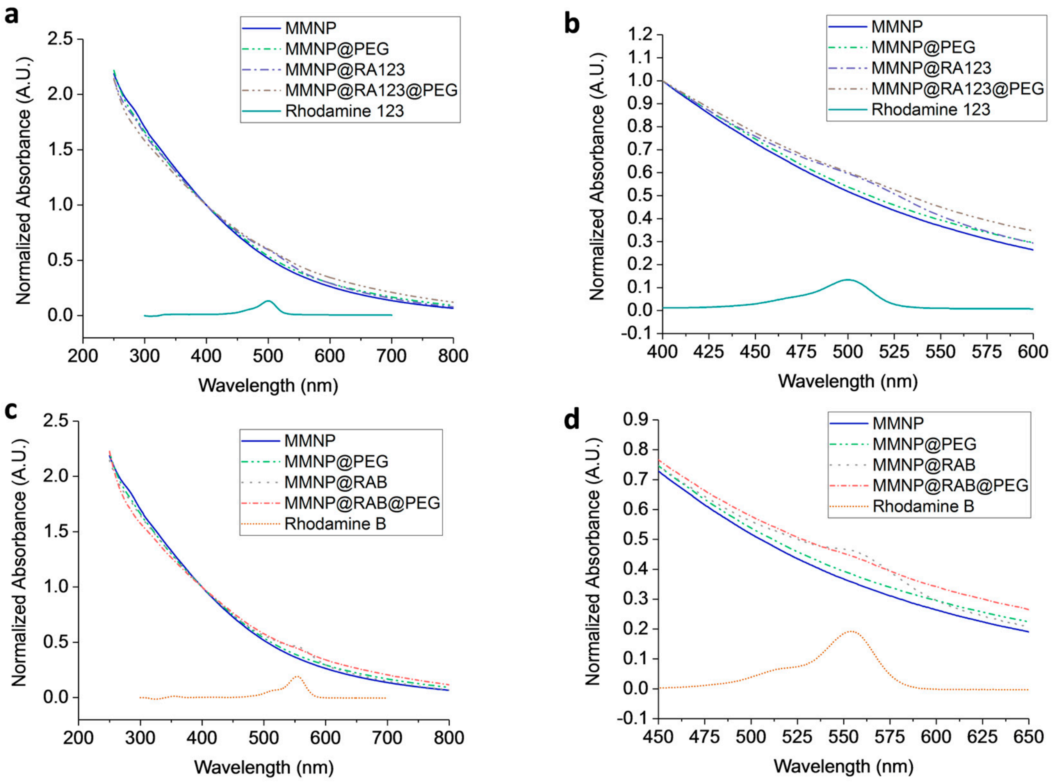

Figure S8. Ultraviolet–visible absorbance spectra of in situ labeled MMNPs. (**a**–**b**) A small absorbance peak is observed in the spectrum of MMNP@RA123 at λ = 510 nm, but not in the spectrum of MMNP@RA123@PEG. This peak is slightly red-shifted from that of free RA123 (λ = 500 nm). (**c**–**d**) A small absorbance peak appears in the spectrum of MMNP@RAB at λ = 560 nm, but not in that of MMNP@RAB@PEG. The absorbance peak of free RAB is at λ = 554 nm. A.U.: Arbitrary units.

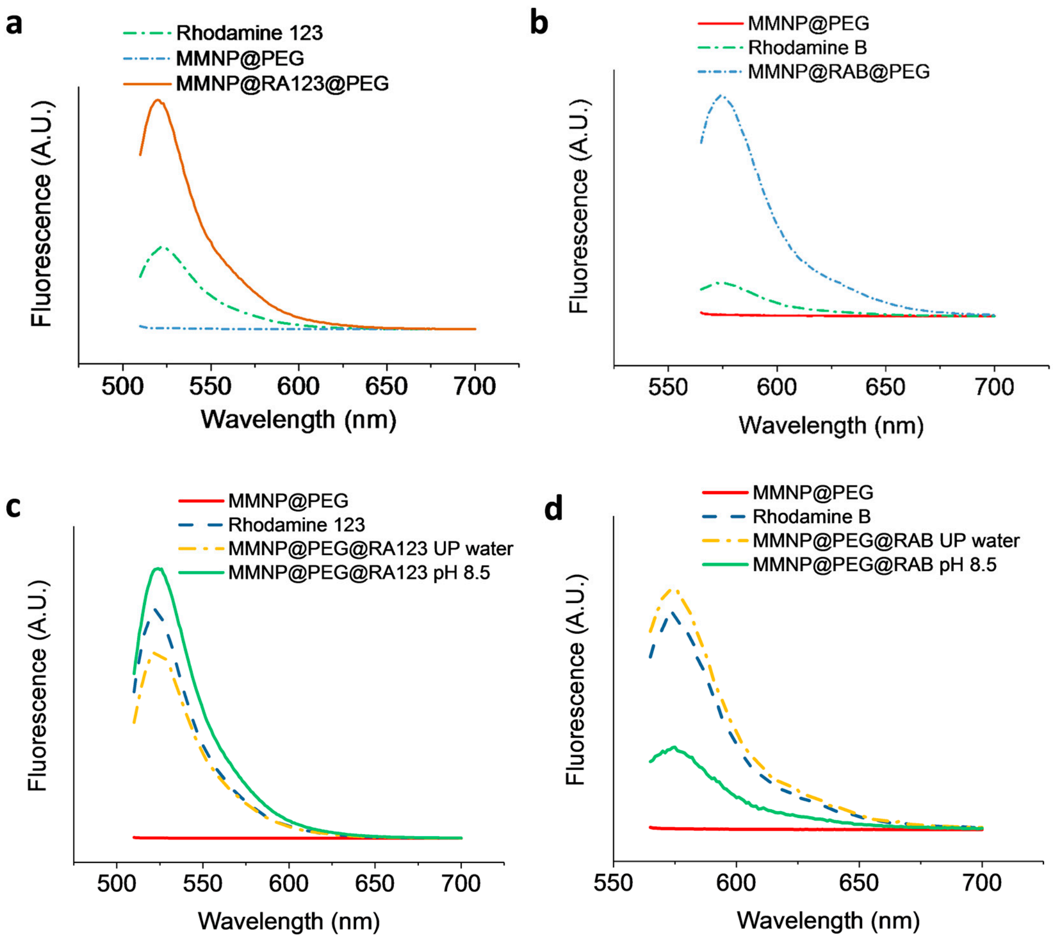

Figure S9. Fluorescence emission spectra of rhodamine 123- and rhodamine B-labeled MMNP samples following centrifugal filtration and before dialysis. (**a**–**b**) Samples prepared by in situ labeling approach with (**a**) rhodamine 123 (MMNP@RA123@PEG) and (**b**) rhodamine B (MMNP@RAB@PEG). (**c**–**d**) Samples prepared by post-functionalization of MMNP@PEG with (**c**) RA123 and (**d**) RAB in either ultrapure water or pH 8.5 buffer. Spectra of 10 ng mL^{-1} pure RA123 or RAB are provided for comparison. A.U.: Arbitrary units.

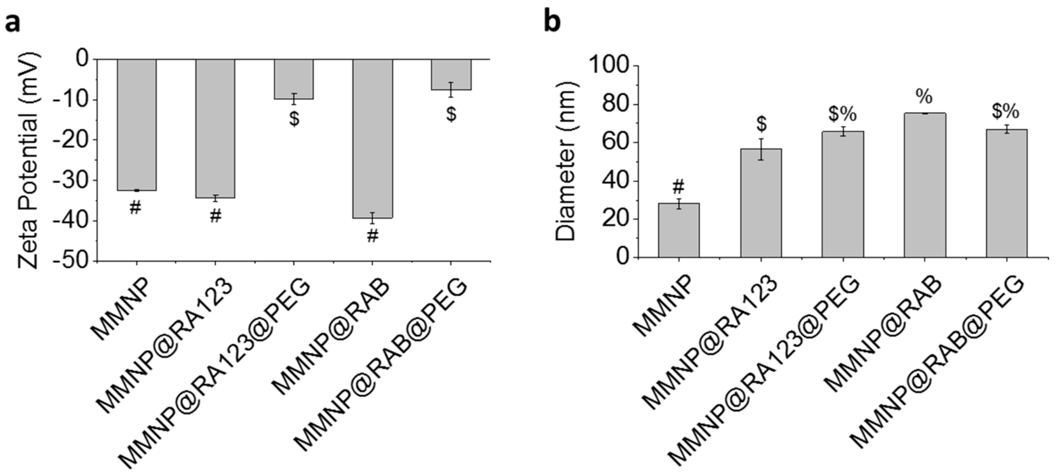

Figure S10. Zeta potential and dynamic light scattering characterization of in situ labeled MMNPs. (**a**) Zeta potentials and (**b**) hydrodynamic diameters of in situ labeled MMNPs before and after PEGylation vs. non-fluorescent MMNPs. Groups not sharing symbols have significantly different values ($p < 0.05$).

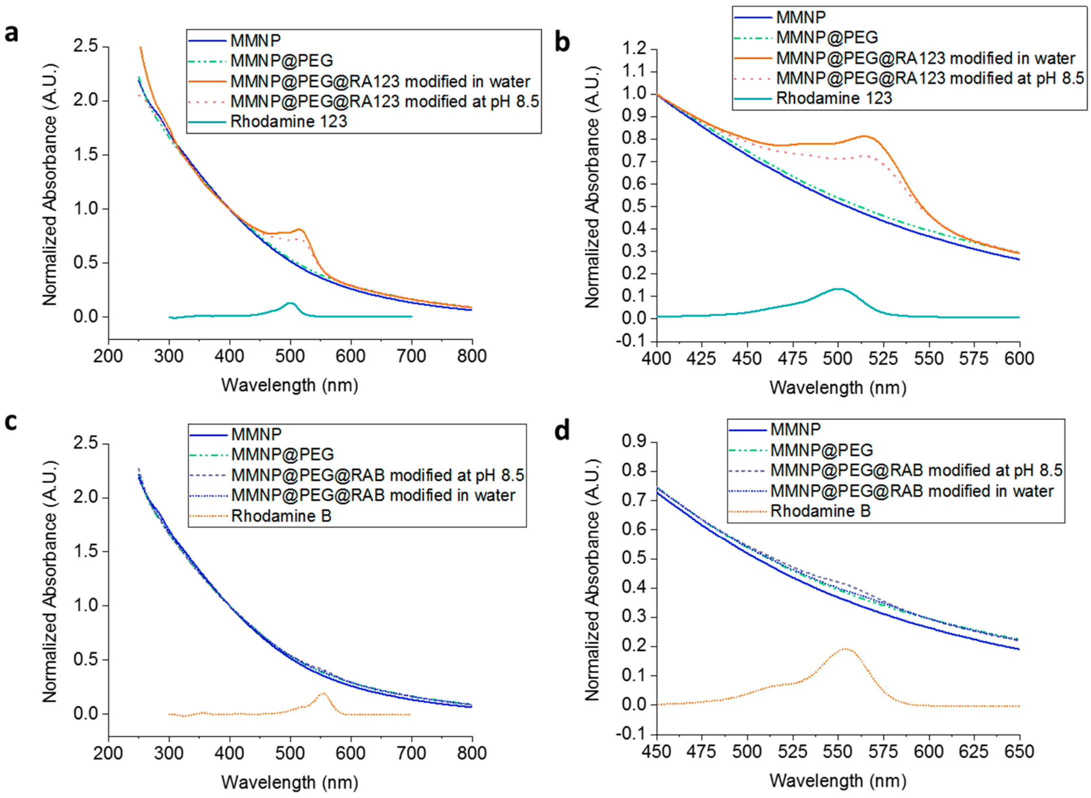

Figure S11. Ultraviolet–visible absorbance spectra of rhodamine-treated MMNP@PEG. (**a**–**b**) A small absorbance peak $\lambda = 520$ nm is observed in the spectrum of MMNP@PEG@RA123 modified in either water or at pH 8.5. This peak is red-shifted from that of free RA123 ($\lambda = 500$ nm). (**c**–**d**) A small absorbance peak at $\lambda = 555$ nm appears in the spectrum of MMNP@PEG@RAB modified at pH 8.5, but not in that of MMNP@PEG@RAB modified in water. The absorbance peak of free RAB is at $\lambda = 554$ nm. A.U.: Arbitrary units.

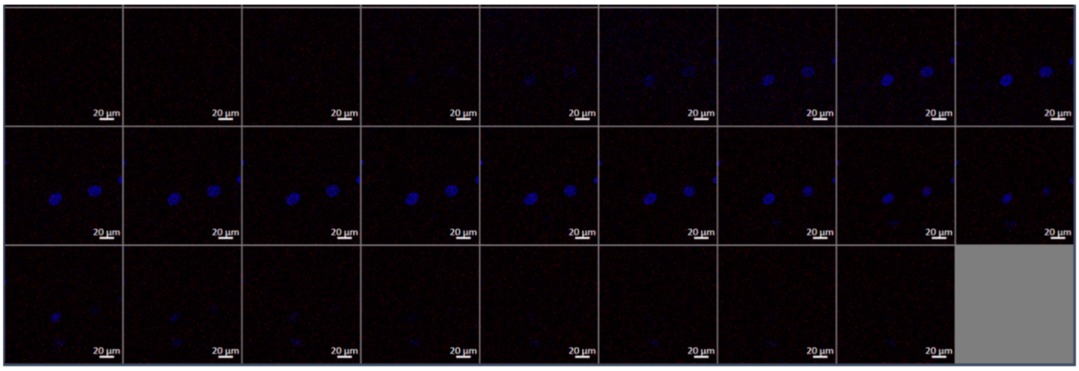

Figure S12. Confocal *z*-stack images of control Hoechst-stained NIH/3T3 fibroblasts untreated with MMNP@PEG@RA123. Focal planes of images are evenly spaced within a 15.3 µm *z*-stack height. Images consist of the merged Hoechst (blue) and RA123 (red) channels. Scale bar: 20 µm.

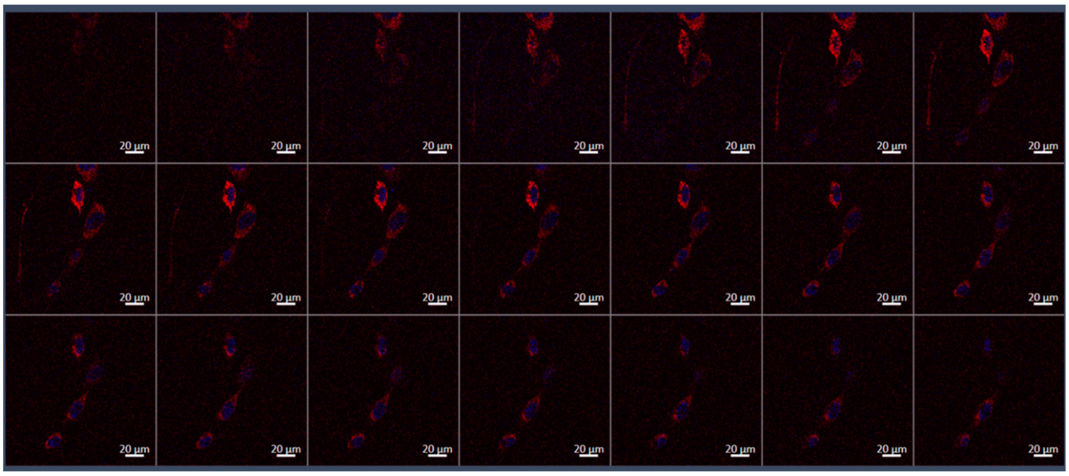

Figure S13. Confocal *z*-stack images of Hoechst-stained NIH/3T3 fibroblasts treated for 24 h with 20 µg mL^{-1} MMNP@PEG@RA123. Focal planes of images are evenly spaced within a 8.3 µm *z*-stack height. Images consist of merged Hoechst (blue) and RA123 (red) channels. Scale bar: 20 µm.

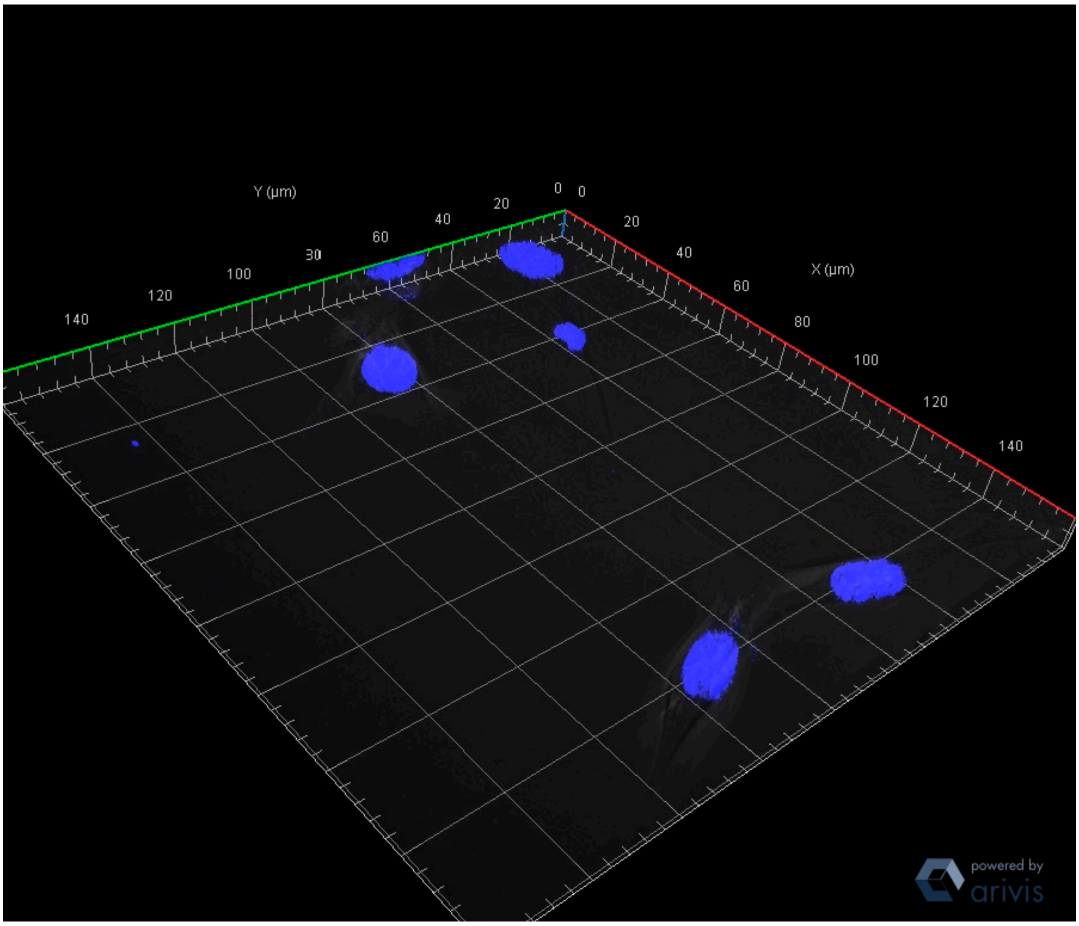

Figure S14. Three-dimensional reconstruction of confocal *z*-stack images of control Hoechst-stained NIH/3T3 fibroblasts untreated with MMNP@PEG@RA123.

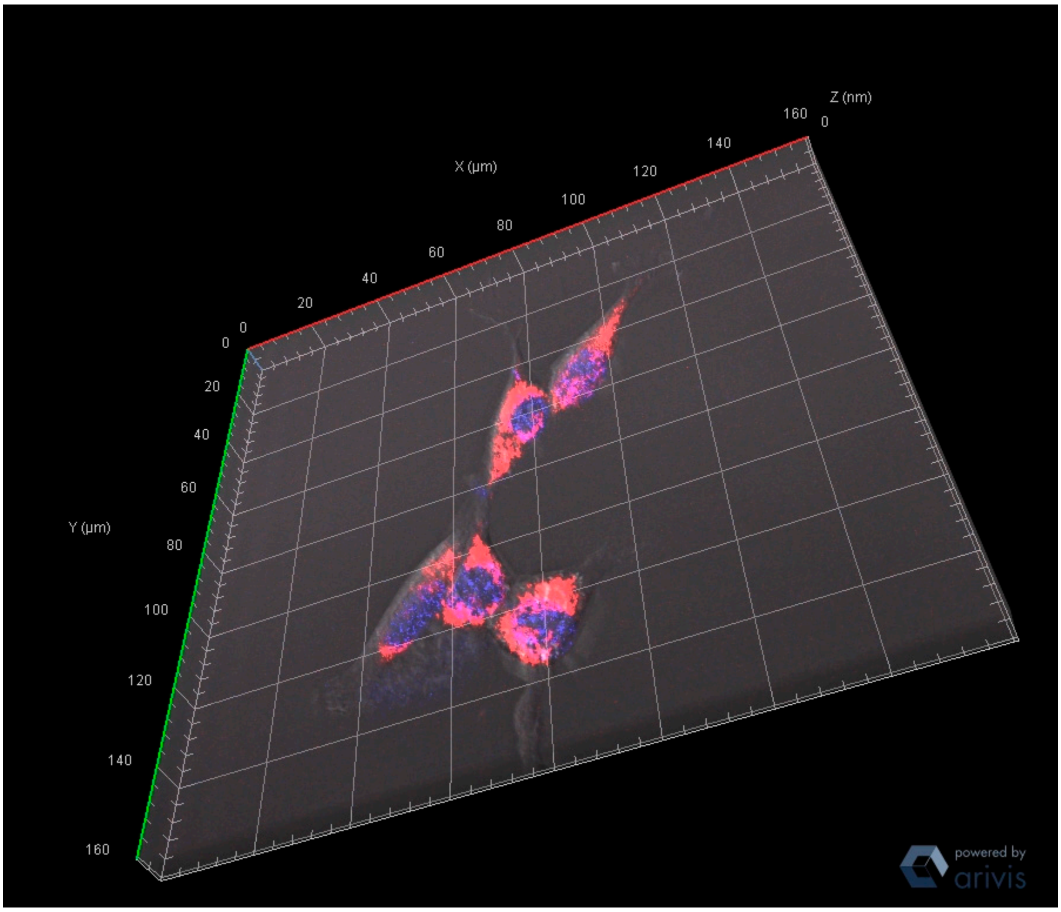

Figure S15. Three-dimensional reconstruction of confocal *z*-stack images of Hoechst-stained NIH/3T3 fibroblasts treated for 24 h with 20 µg mL^{-1} MMNP@PEG@RA123.

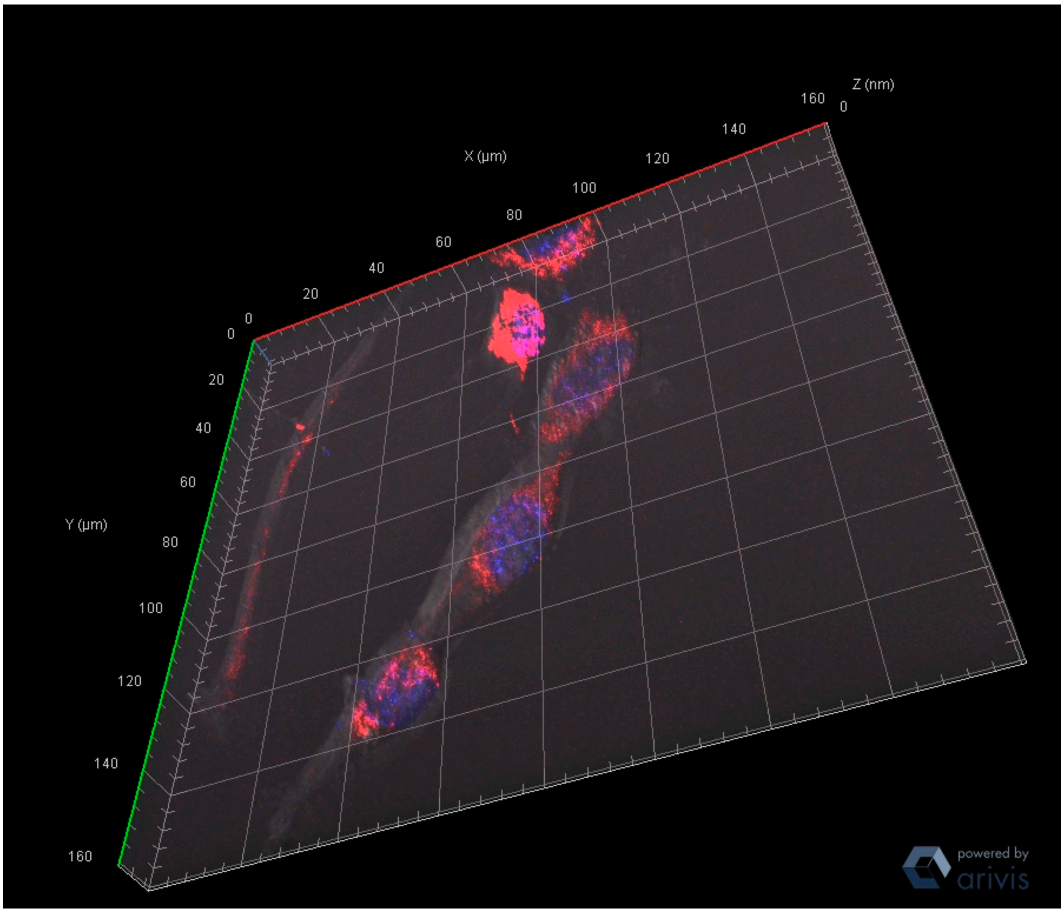

Figure S16. A second area covered by three-dimensional reconstruction of confocal *z*-stack images of Hoechst-stained NIH/3T3 fibroblasts treated for 24 h with 20 µg mL^{-1} MMNP@PEG@RA123.

Table S1. Parameters for exponential decay functions fitting of extinction coefficient vs. wavelength in Supplementary Figure S1.

$$\varepsilon(\lambda) = Ae^{\left(-\frac{\lambda}{b}\right)} + C$$

Synthesis Condition	*A*	*b*	*C*	r^2
2 mg mL^{-1} DA, 0.8:1 NaOH:DA (121 nm)	0.1571	215.1	-1.34×10^{-4}	0.9992
2 mg mL^{-1} DA, 0.9:1 NaOH:DA (88 nm)	0.1489	206.5	6.83×10^{-5}	0.9980
2 mg mL^{-1} DA, 1:1 NaOH:DA (47 nm)	0.1319	196.9	6.83×10^{-5}	0.9984
1 mg mL^{-1} DA, 1:1 NaOH:DA (26 nm)	0.1197	202.7	3.01×10^{-5}	0.9989

Mean extinction coefficient values at each wavelength shown from 300 to 999 nm were fit to the exponential decay function shown above. Average batch-to-batch D_h of nanoparticles used to calculate extinction coefficients are shown. λ: Wavelength (nm); ε: Extinction coefficient (absorbance units × cm^{-1} × (µg/mL)$^{-1}$); A, C: Fitting parameters (absorbance units × cm^{-1} × (µg/mL)$^{-1}$); b: Fitting parameter (nm^{-1}).

Table S2. Comparison of MMNP size distributions calculated by DLS and cryo-TEM for the samples analyzed in Figure 3d–i.

**Synthesis Conditions**		**DLS**	**Cryo-TEM**	
Dopamine·HCl Concentration	NaOH:DA (mol:mol)	Mean MMNP Diameter (nm)	Mean MMNP Diameter (nm)	σ (nm)
2 mg mL^{-1}	0.8:1	139.5	89.7	26.8
2 mg mL^{-1}	1:1	52.1	60.0	12.4
1 mg mL^{-1}	1:1	21.7	26.6	9.1

Nanoparticle diameters calculated by these methods are within 10 nm of one another for both samples prepared at 1:1 NaOH:DA, but deviate significantly for the sample prepared at 2 mg mL^{-1} DA with 0.8:1 NaOH:DA.

References

1. Sarna, T.; Swartz, H.A. The physical properties of melanins. In *The Pigmentary System*; Blackwell Publishing Ltd.: Hoboken, NJ, USA, 2007; pp. 311–341.
2. Sarna, T.; Sealy, R.C. Photoinduced oxygen consumption in melanin systems. Action spectra and quantum yields for eumelanin and synthetic melanin. *Photochem. Photobiol.* **1984**, *39*, 69–74, doi:10.1111/j.1751-1097.1984.tb03406.x.
